# A miR172 target-deficient AP2-like gene correlates with the double flower phenotype in roses

**DOI:** 10.1038/s41598-018-30918-4

**Published:** 2018-08-27

**Authors:** Léa François, Marion Verdenaud, Xiaopeng Fu, Darcy Ruleman, Annick Dubois, Michiel Vandenbussche, Abdelhafid Bendahmane, Olivier Raymond, Jérémy Just, Mohammed Bendahmane

**Affiliations:** 1 Laboratoire Reproduction et Développement des Plantes, Univ Lyon, ENS de Lyon, UCB Lyon 1, CNRS, INRA, F-69364 Lyon, France; 2 Institute of Plant Sciences Paris-Saclay IPS2, CNRS, INRA, University Paris-Sud, University of Evry, University Paris- Diderot, Sorbonne Paris-Cite, University of Paris-Saclay, F-91405 Orsay, France; 3 Key Lab of Horticultural Plant Biology, College of Horticulture & Forestry Sciences, Huazhong Agricultural University, Wuhan, China

## Abstract

One of the well-known floral abnormalities in flowering plants is the double-flower phenotype, which corresponds to flowers that develop extra petals, sometimes even containing entire flowers within flowers. Because of their highly priced ornamental value, spontaneous double-flower variants have been found and selected for in a wide range of ornamental species. Previously, double flower formation in roses was associated with a restriction of *AGAMOUS* expression domain toward the centre of the meristem, leading to extra petals. Here, we characterized the genomic region containing the mutation associated with the switch from simple to double flowers in the rose. An *APETALA2*-*like* gene (*RcAP2L*), a member of the Target Of EAT-type (TOE-type) subfamily, lies within this interval. In the double flower rose, two alleles of *RcAP2L* are present, one of which harbours a transposable element inserted into intron 8. This insertion leads to the creation of a miR172 resistant *RcAP2L* variant. Analyses of the presence of this variant in a set of simple and double flower roses demonstrate a correlation between the presence of this allele and the double flower phenotype. These data suggest a role of this miR172 resistant *RcAP2L* variant in regulating *RcAGAMOUS* expression and double flower formation in *Rosa* sp.

## Introduction

Roses are widely used as garden ornamental plants and cut flowers worldwide. A number of their agricultural and decorative traits specify their commercial value^1^ and have been selected during domestication. Examples of these important traits are recurrent flowering, double flowers, petal colour and fragrance^2^. Double flower refers to a characteristic of modern roses giving blooms with an increased number of petals that can vary from 10 to more than 200 petals per flower, whereas wild-type simple flowers are composed of 5 petals. This characteristic is tightly associated with flower development and organ identity patterning, as it results from homeotic conversion of stamens into petals^3^. However, the underlying molecular mechanisms are not fully understood in roses, or in other non-model species.

In the past three decades, most of the genetic and molecular networks controlling floral development have been extensively studied in model species such as *Arabidopsis thaliana* and *Antirrhinum majus*. These studies led to the establishment of the ABCE model of flower development^4,5^. In this model, the combinatorial actions of four classes of homeotic genes (A, B, C and E) determine flower organ identity. Briefly, from the outer to the inner whorl of the floral meristem, the A-class genes (*APETALA1*, *AP1*; *APETALA2*, *AP2*) alone determine sepal formation; the A-class genes together with the B-class genes (*PISTILLATA*, *PI*; *APETALA3*, *AP3*) determine petal fate, the C-class gene (*AGAMOUS*, *AG*) associated with the B-class genes specify stamen formation, and finally the C-class gene determines carpel fate. E-class genes are necessary for all floral organ identity. A-class genes have also an antagonistic role toward the expression of the C-class gene *AG*, and vice versa. This leads to the expression of the A-class genes in the sepal and petal whorls and of *AG* in the stamen and carpel whorls. In *Arabidopsis*, *AG* loss-of-function leads to over-accumulation of A-class genes in the third whorl and homeotic conversion of stamens into petals^6^. Similarly, over-accumulation of AP2 protein leads to a reduced expression of *AG* in the third whorl and a similar homeotic conversion of stamens into petals^7^. This conceptual framework for floral organ identity patterning is broadly valid for flowering plant species that have been studied^8,9^. However, during evolution, some genes underwent duplication and neo- or sub-functionalization, leading to small differences in their regulatory interactions. For example, the canonical C-function, performed by *AG* in *Arabidopsis*, is carried out by *PLENA* in *Antirrhinum*, that is orthologous to the *Arabidopsis* *SHATTERPROOF* genes (*SHP*)^10^. In *Petunia*, the restriction of the C-class gene expression needs mainly the actions of the microRNA *BLIND*, but involves a gene from the euAP2 family, *PhBEN*^11,12^. This diversity of the canonical ABCE functions, together with the absence of comprehensive genome data giving access to all members of each gene family, hampered the identification of the key genes determining floral organ identity in non-model species, such as in rose. Recently, efforts have been made to identify canonical rose A-, B-, C- and E-class gene orthologues, but we are still far from understanding their exact role in rose floral phenotype patterning^3,13–19^.

Previously, we demonstrated that a downregulation and a restricted expression domain of the rose orthologue of *AGAMOUS* (*RcAG*) correlates with an increase in petal number in domesticated roses^3^. This was later confirmed by transient *RcAG* downregulation using Virus Induced Gene Silencing^20^. Similar associations between *AG* expression and double flowers formation were shown in other species such as Ranunculids, Cyclamen, Japanese gentian and *Prunus*^21–24^. Yet, the molecular mechanism by which the restriction of the expression of *RcAG* occurs remains unknown. Indeed, the rose *RcAG* gene does not co-segregate with the major locus (*Df*) located on Linkage Group 3 that has been shown to control the switch from the simple flower to the double flower phenotype^25,26^. In roses, a yet unknown gene located in the *Df* locus and acting upstream of *RcAG* must be the determinant for double flower formation^3^.

In order to identify the genetic determinant of the double flower phenotype, we localized and analysed the sequence of the double flower interval using the recent high-quality *Rosa chinensis* ‘Old Blush’ genome assemblies^27^. The first corresponds to the homozygous rose assembly^27,28^ consisting of seven assembled pseudomolecules and representing a haplotype of the rose genome. The second assembly corresponds to the heterozygous *Rosa chinensis* ‘Old Blush’ consisting of 15,937 scaffolds, and provides access to the two haplotypes of the genome and information on alleles. Among the candidate genes in the interval, we identified a gene belonging to the euAP2 family, of which certain members are known to repress *AG* expression in many species^6,12,29,30^. We show that in double flower roses this gene is present as two different alleles, one of which harbours a transposable element insertion that is never found in simple flower roses. This insertion leads to a truncated *RcAP2L* version that lacks the miR172 binding site, meaning it is no longer negatively regulated by this microRNA. The data provide a basis for a mechanism by which double flowers are formed and open new perspectives to dissect in detail the underlying molecular and biochemical mechanisms in roses and likely in other species.

## Results

### Localization of the genetic interval associated with the double flower phenotype

**Figure 1:**
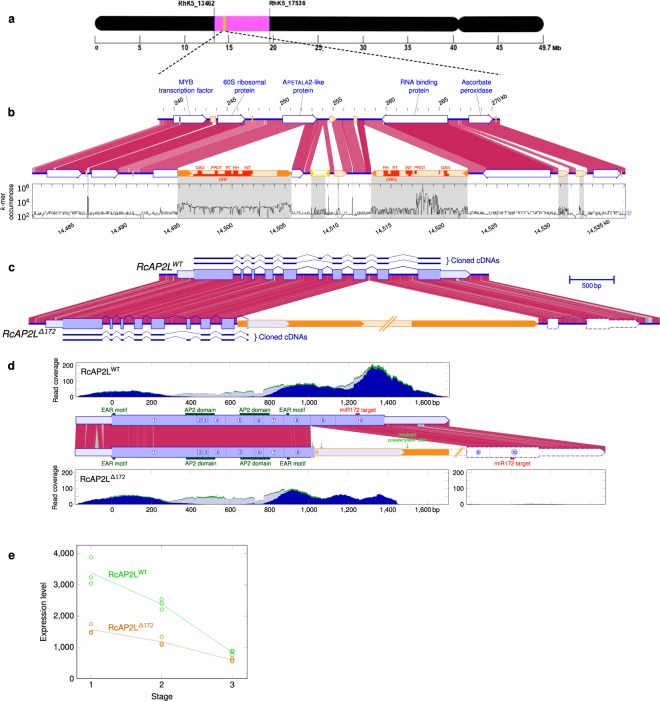
*RcAP2L* lies within the double flower interval in the rose. (**a**) Graphical representation of chromosome 3 showing the localization of the double flower interval and its flanking markers from the genetic map. (**b**) Detailed comparison of the two haplotypes containing *RcAP2L* and neighbouring sequences. The upper haplotype contains the wild-type allele of *RcAP2L*. The lower haplotype contains *RcAP2L*^*∆172*^ with TE insertion. Blue and orange box-arrows indicate annotated genes and transposable elements, respectively. Sequence similarities between the two haplotypes are shown with purple shades. The darker is the purple colour intensity, the highest is the Blastn identity (ranging between 100 to 90%). The plot shows *k*-mer occurrences along the region: corresponds to the number of times each word of 47 bp in length is found in the 375 Gb set of reads used to assemble the heterozygous genome^27^. High occurrence counts indicate repeated sequences (grey shading). (**c**) Intron/exon structure of the two alleles of *RcAP2L*, confirmed by cDNA sequencing. Exons are shown as blue boxes. Light blue indicate 5′ and 3′ UTR. cDNA sequences confirmed by RT-PCR are shown. The transposable element inserted in *RcAP2L*^*∆172*^ is shown in orange. Purple lines show identical sequence and grey lines indicate single nucleotide polymorphisms and short INDELs (overall identity: 93,4%). (**d**) Structure of mature mRNAs from *RcAP2L* alleles. The three area plots show RNA-seq read coverage along mRNAs. Dark blue: reads matching specifically one of the two alleles. Light blue: reads that could come from any of the two *RcAP2L* alleles. Green: reads also matching at other loci in rose genome. Expression data confirm the structure of the two alleles, including the correct splicing of exon 9′ of *RcAP2L*^*∆172*^ originating from the inserted TE, and the appearance of a new polyadenylation site at position 1,500 bp. The region of *RcAP2L*^*∆172*^ homologous to exons 9 and 10 exhibits a negligible expression level (highest value ≤ 2). (**e**) Expression of wild-type allele *RcAP2L*^*WT*^ (green) and *RcAP2L*^*∆172*^ allele (orange) early on in flower formation. Stage 1: sepal initiation, stage 2: petal initiation, stage 3: stamen initiation. Data were extracted from RNA-seq analyses. Y-axis is labelled in FPKM.

In roses, the double flower phenotype is associated with a dominant mutation in the yet unknown *Df* (*DOUBLE FLOWER*) locus. This locus was previously shown to map on LG3^25,26^. We used the high-quality genome assembly (RcHm)^27,28^ to identify flanking markers that define the mapping interval containing *Df*. Flanking markers were retrieved from the previously reported genetic maps^26,31,32^ and mapped on the rose genome sequence^27^ and those that had unique match allowed to mark out an interval of 6.2 Mb on Chromosome 3 at coordinates 13,535,933 to 19,743,495 (Fig. 1a). Genes within this interval were then retrieved using the gene annotation of the reference rose genome^27,28^ (Supplementary Table 1). The assembled interval on Chromosome 3 contained 631 annotated genes (Supplementary Table 1). Alleles for each gene were then retrieved using the genome assembly of the heterozygous genome (RcHt)^27^. Previous studies showed that a modified expression pattern of *RcAG* was associated with double flower formation in rose^3^. *RcAG* maps on Chromosome 5 of the rose genome, thus corroborating previous data indicating that *RcAG* is not the *Df* gene^3^. Among the 631 annotated genes that lie within the double flower mapping interval, no gene showed similarities to *RcAG* gene. These data suggest that the gene responsible for double flower formation could be an upstream regulator of *RcAG*.

### A mutant allele of an AP2-like gene lies within Double Flower interval

To narrow down the number of *Df* gene candidates, we searched within the assembled double flower interval for genes that share homologies with those known to regulate *AG* expression in *Arabidopsis* and that are present at heterozygous state in the double flower rose *R. chinensis* ‘Old Blush’. Indeed, previous genetic segregation analyses involving “Old Blush” or other rose cultivars as parents showed that the double flower trait is controlled by a dominant allele at heterozygous state^25,33–35^. Interestingly, one candidate gene had high sequence similarity to *APETALA2* (*AP2*). In *Arabidopsis*, *AP2* was shown to negatively regulate the expression of *AG* in the sepal and petal whorls, restricting its expression to the stamen and carpel whorls^6,36^. The identified rose *AP2-like* gene (*RcAP2L*, *RcHm3g0468481*; Fig. 1c) contains 10 exons and 9 introns, and encodes for a 460 amino-acid protein. Analysis of the predicted RcAP2L protein showed the presence of two AP2 DNA-binding domains, indicating a similar structure to the *Arabidopsis* A-class gene *AP2*^37^. Additionally, a miR172 binding site and two EAR motifs (Ethylene-responsive element binding factor-associated amphiphilic repression) were also found in this gene. These three features are characteristic of the euAP2 family members^38,39^.

**Figure 2:**
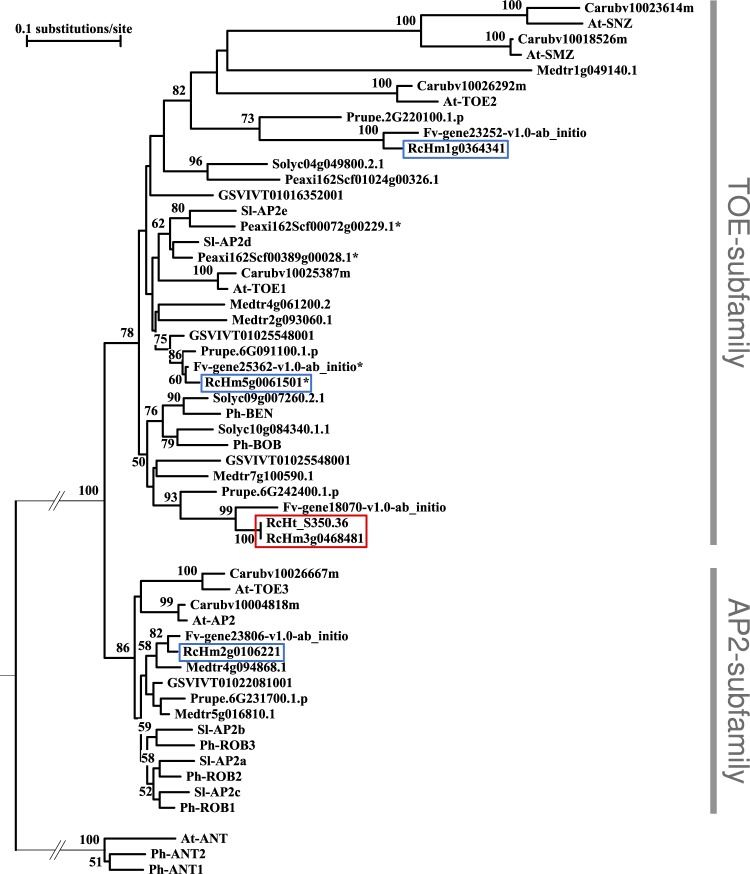
*RcAP2L* belongs to the euAP2 family and the TOE-subfamily. Neighbor-Joining tree based on the aligned AP2 DNA binding domains of the euAP2 members from *Rosa chinensis* (RcHm and RcHt)^27^, *Fragaria vesca* (Fv), *Petunia hybrida* and *P*. axillaris (Ph and Peaxil, respectively), *Solanum lycopersicum* (Solyc or Sl), *Arabidopsis thaliana *(At), *Capsella rubella* (Carub), *Medicago truncatula* (Medtr), *Vitis vinifera* (GSVIVT) *and Prunus persica* (Prupe). The tree was rooted with *AINTEGUMENTA* (*ANT*) and tested with 2,000 bootstraps. EuAP2 members clearly group together with a bootstrap of 100. Among this family, the AP2- and TOE-subfamilies are also well separated (bootstrap of 86 and 78 respectively). Each distinct gene of close species generally groups together confirming the reliability of the tree. *RcAP2L* is part of the TOE-subfamily (observed for both the truncated  *RcHm3g0468481* and the wild-type *RcHt_S350.36* version).

BLASTP of the *Arabidopsis* AP2 protein on rose and strawberry predicted protein sequences identified four potential members of the euAP2 family in each of the species (Fig. 2). Protein sequence alignments and phylogenetic analyses using the AP2 domains of euAP2 genes from *Petunia hybrida*, *Solanum lycopersicum, Arabidopsis thaliana, Capsella rubella, Medicago truncatula, Vitis vinifera* and *Prunus persica* showed that each of the four rose predicted proteins groups with a single and unique strawberry and *Prunus* predicted protein, supporting their orthologous relationship and the quality of the tree (Fig. 2). Bootstrap values highly support the presence of a single rose member of the AP2-type subfamily (*RcHm2g0106221*). The remaining three rose euAP2 members, including *RcAP2L*, likely belong to the Target Of EAT-type (TOE-type) subfamily (Fig. 2). The rose TOE-type subfamily contains a single homolog for *AtTOE1* (*RcHm5g0061501*) and a gene (*RcHm1g0364341*) that groups in a branch with *Arabidopsis TOE2*, *SMZ* (*SCHLAFMUTZE*) and *SNZ* (*SCHNARCHZAPFEN*). The higher divergence of this last branch from the rest of the tree is likely due to the presence of a non-functional second AP2 DNA-binding domain, that could have accumulated more mutations and putatively acquired a new function^12,39^.

Phylogenetic analyses, using AP2 domains, revealed no direct orthologue of *RcAP2L* (*RcHm3g0468481*) in *Arabidopsis* genome. Interestingly, *RcAP2L* appears to group with the *Petunia PhBEN* and *PhBOB* gene. *PhBEN* was reported to repress the expression of the C-function genes in the perianth, and together with *PhBOB*, it is required for organ growth in the second whorl^12^.

Gene sequence analyses, using the assembled heterozygous genome of ‘Old Blush’, revealed that in the double flower of *R. chinensis* ‘Old Blush’, *RcAP2L* is present as two different alleles. The first allele, located on scaffold *RcHt_S350*^27^, corresponds to the wild-type sequence of *RcAP2L* (*RcAP2L*^*WT*^). A second allele, located on two assembled scaffolds (*RcHt_S3277* and *RcHt_S1251*), contains an additional sequence of 10,790 bp inserted in its 8^th^ intron (genome coordinates 14,494,849 – 14,505,638; Fig. 1b).

The inserted sequence is repeated in the rose genome and corresponds to a transposable element (TE) belonging to the Gypsy LTR retrotransposon family (Fig. 1b). Sequence alignment also showed that the two LTRs of the inserted TE are 100% identical on their whole length, indicating a recent insertion^40^. The inserted TE contains an open reading frame of 5,535 pb and DANTE software predicted the presence of at least 5 retroviral sequences coding for the structural protein GAG, a protease, a reverse transcriptase, a H-Ribonuclease and an integrase. We found 17 complete copies from this TE family in the rose genome, and 66 solo-LTRs, making it moderately repeated.

The TE insertion in *RcAP2L* creates a new splicing acceptor site that is predicted to lead to a fusion of the 8^th^ exon of *RcAP2L* with a sequence from the 5′ LTR from the TE. This new splicing creates a premature STOP codon and the loss of the 9^th^ and the 10^th^ exons, which causes the formation of a truncated protein composed of 342 amino acids, and the loss of the miR172 binding site (Fig. 1d). This allele was consequently named *RcAP2L*^*∆172*^.

We mapped RNA-seq reads on the predicted transcripts to validate the mRNA structures; as a few SNPs and INDELs exist between the sequences of the two alleles (Fig. 1d), we were able to distinguish reads coming from each. The RNA-seq coverage drastically decreased at one of the predicted polyadenylation sites of *RcAP2L*^*∆172*^ identified by PASPA software^41^, indicating that the corresponding mRNA existed and was properly spliced, and thus must be stable. The RNA-seq mapping also showed that exons 9 and 10 of the mutated allele, located after the TE insertion, are not expressed, indicating that the mRNA from this allele no longer have a miR172 binding site. Sequencing of cDNA prepared from RNA extracted from *R. chinensis* ‘Old Blush’ confirmed the predicted intron/exon structures but also indicated a potential alternative splicing with the loss of the 6^th^ exon (Fig. 1c).

Expression analyses showed that both alleles *RcAP2L*^*WT*^ and *RcAP2L*^*∆172*^ are expressed during flower formation (Fig. 1e). The expression of both alleles is high in flower primordia at stages 1 and 2 (sepal and petal initiation, respectively), and their expression starts to significantly decrease at stage 3 (stamen initiation), thus consistent with a role in perianth formation.

### The presence of the *RcAP2L*^*∆172*^ allele correlates with double flower formation in Chinese and modern roses

To further address the correlation between the presence of the *RcAP2L*^*∆172*^ allele and double flower formation, we investigated its presence in the available genomic data from five other rose genotypes that exhibit either double flowers (*R. odorata* ‘Hume’s Blush’, *R. x hybrida* ‘La France’) or simple flowers (*R. chinensis* ‘Sanguinea’, *R. chinensis* ‘Spontanea’ and *R. wichurana*)^27^. The insertion of the TE in intron 8 of *RcAP2L* was investigated by the presence of reads overlapping both 5′ and 3′ junctions, while its absence was confirmed by reads overlapping the intact position on the wild type *RcAP2L* gene (Table 1). For example, a mean of 6.9 reads per 10^8^ reads and 9.2 reads per 10^8^ reads were shown to overlap respectively the 3′ and 5′ TE junctions in *R. odorata* ‘Hume’s Blush’ and 8.4 reads per 10^8^ reads were overlapping the wild type position of the gene (Table 1), indicating that this genotype had one of each allele. Conversely, *R. wichurana* had no read overlapping the TE junctions and 8.6 reads per 10^8^ reads overlapping the intact position, indicating that this genotype only has the wild type allele at homozygous state. This analysis indicated that all double flower roses of the panel harbour both the wild-type *RcAP2L*^*WT*^ allele and the truncated *RcAP2L*^*∆172*^ allele. Conversely, simple flower roses harbour only the *RcAP2L*^*WT*^ allele and never the *RcAP2L*^*∆172*^. Together, these data corroborate the observation in ‘Old Blush’ and show the existence of a correlation between the presence of *RcAP2L*^*∆172*^ and double flower formation.

**Table 1:** Identification of RcAP2L alleles present in 7 resequenced genotypes.

Genotype	Flower multiplicity	Ploidy level	Overlapping reads per 10^8^ reads		
			WT	5′ of TE	3′ of TE
*R. chinensis* ‘Old Blush’	Double flower	2x	5.7	7.2	7.4
*R. chinensis* homozygous genome	NA	2x	0	22.2	16.7
*R. odorata* ‘Hume’s Blush’	Double flower	2x	8.4	9.2	6.9
*R*. x *hybrida* ‘La France’	Double flower	3x	5.1	5.1	8.0
*R. chinensis* ‘Sanguinea’	Simple flower	2x	22.5	0	0
*R. chinensis* ‘Spontanea’	Simple flower	2x	13.2	0	0
*R. wichurana*	Simple flower	2x	8.6	0	0

Number of 100 bp genomic reads overlapping intra-gene (denoting the presence of a wild-type *RcAP2L* allele) or gene-TE (mutated allele, *RcAP2L*^*∆172*^) junctions, for genotypes with double or simple flowers. The read counts were normalized according to the read library size, and expressed as reads per 100 million reads. Ploidy level: According to Raymond *et al*.^27^.

**Figure 3:**
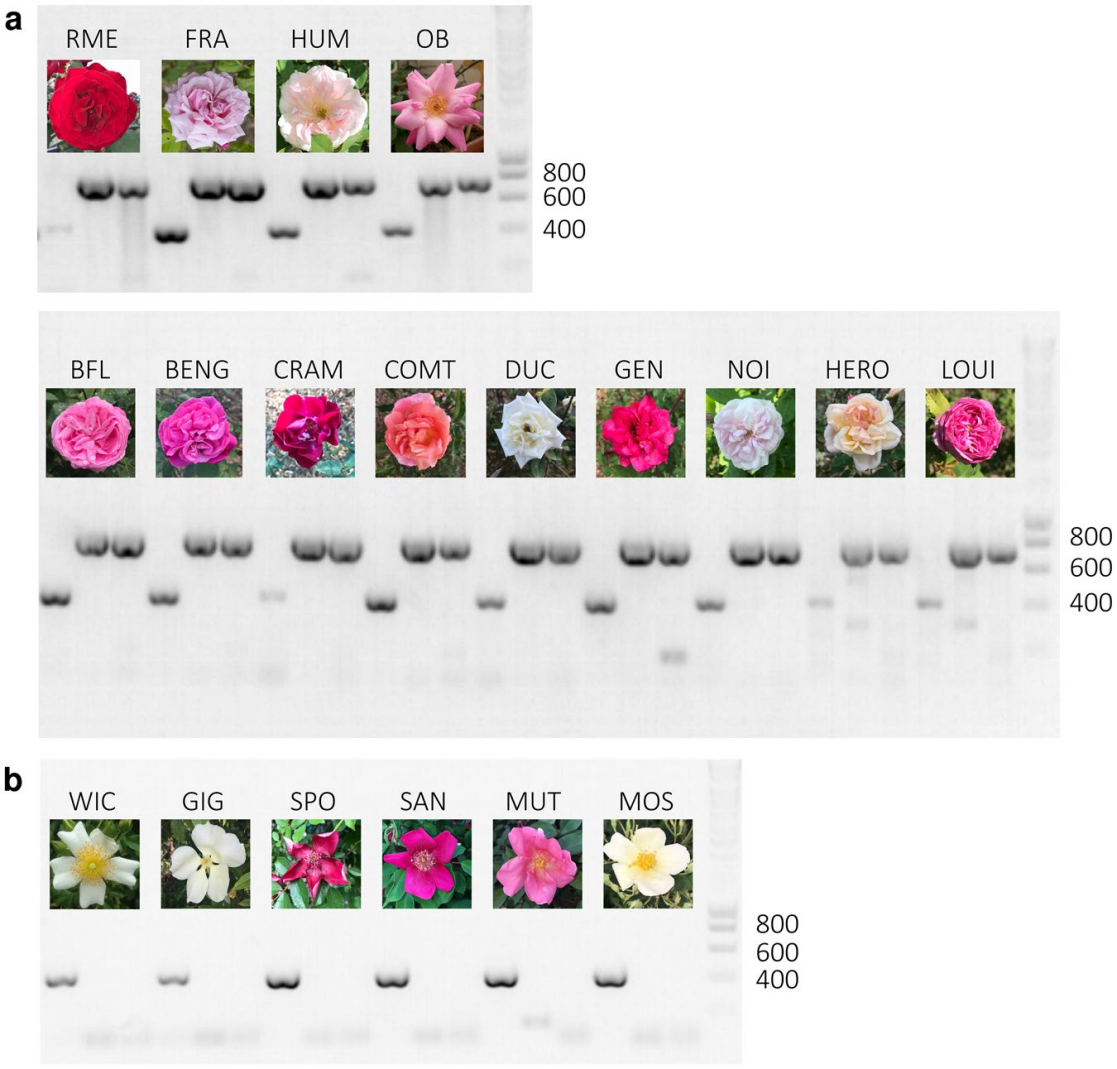
The transposable element insertion is only observed in the analysed double flower roses. PCR to detect the TE were performed on genomic DNA from different rose cultivars exhibiting simple (**b**) or double flowers deriving from *R. chinensis* (**a**). The lower band (419 bp) corresponds to the combination of primers that amplifies the wild type allele, while the two higher ones (770 bp and 754 bp) correspond to amplification of the left and right borders of the transposable element insertion, respectively.

To further confirm our hypothesis, we investigated the presence of *RcAP2L*^*∆172*^ in a set of modern rose cultivars (Supplementary Table S2). The presence or absence of *RcAP2L*^*∆172*^ was investigated by PCR amplification of the TE insertion junctions using DNA extracted from 6 rose plants exhibiting simple flowers and from 13 rose plants exhibiting double flowers (Supplementary Table 2; Fig. 3). DNA fragments overlapping both left and right borders of the transposon were detected in all these double flower roses (Fig. 3a), while no similar DNA fragment could be detected in the simple flower roses (Fig. 3b). Our data show a correlation between the double flower phenotype and the presence of the transposable element insertion, thus providing another argument in favour of the role of *RcAP2L*^*∆172*^ during double flower formation. It should be noted that none of the analysed double flower cultivars were homozygous for the truncated allele and all had wild type and truncated alleles.

## Discussion

In roses, the formation of double flowers is associated with a shift of the A/C boundary and a restriction of *RcAG* expression domain toward the centre of the meristem which in turn leads to a conversion of stamens into petals^3^. Genetic mapping identified the major locus *Df* as involved in the control of rose double flower formation^25^. However, *RcAG* does not lie within the double flower interval suggesting that a yet unknown upstream regulator of *RcAG* must be the determinant of double flower formation.

In this study, we identified a gene of the euAP2 family, *RcAP2L*, that localizes within the double flower interval. We identified a truncated allele version of *RcAP2L* (*RcAP2L*^*∆172*^) whose presence correlates with double flower formation in Chinese roses and modern roses, such as *R. hybrida* ‘La France’, that have Chinese rose cultivars as ancestors. Such an allelic form is absent in all simple flower roses. A TE insertion in the 8^th^ intron of *RcAP2L* leads to the loss of the miR172 target site. We demonstrate that the position of the TE insertion is conserved among different double flower rose varieties that have a parent from the Chinense section. These data indicate that this allele must have been inherited from a single common Chinese ancestor and spread among its double flower modern descendants due to the positive human selection during rose domestication.

Phylogenetic analysis indicates that *RcAP2L* is a member of the TOE-subfamily. In *Arabidopsis*, most studies focused on *AP2* and only a few reports addressed the role of other euAP2 family members such as *TOE1*, *TOE2* and *TOE3*. *Arabidopsis AP2* is known to restrict *AG* expression to the third and fourth whorls (stamens and carpels, respectively). Knockout of *AP2* results in ectopic expression of *AG* in the sepal and petal whorls, which is associated with a conversion of sepals to carpeloid structures and loss of petals^6^. Recently, ChIP-qPCR experiments in *Arabidopsis* showed that both AP2 and TOE3 bind to *AG* second intron (containing transcription cis-regulatory elements) to decrease its expression level^29,42^. These published data support our findings on *RcAP2L* as a pertinent candidate for double flower determination, likely by regulating the expression pattern of *RcAG*.

In *Arabidopsis*, *TOE1* overexpression induces late flowering while its loss-of-function leads to early flowering with no apparent flower phenotype variations^43^. Conversely to *Arabidopsis*, in some species certain TOE-subfamily genes have a flower patterning function. In *Petunia*, *PhBEN* was shown to inhibit the C-function in the perianth primordium, thus consistent with a function similar to that of the *Arabidopsis AP2*. It is clear that *TOE* genes have evolved to perform different functions in different species, and in roses, their misexpression is likely at the origin of the appearance of the double flower abnormality.

EuAP2 family members are characterized by the presence of a miR172 binding site that is important for their post-transcriptional regulation. It has been reported that overexpression of miR172 induces early flowering as well as floral defects similar to the ones observed in *ap2* mutants^43^. During flower formation, miR172 is highly expressed in whorls 3 and 4 and targets *AP2* transcripts to prevent its expression in the centre of the meristem, where the stamen- and carpel-identity gene *AG* is expressed^30^. However, in *Arabidopsis* it has been reported that when *AP2* lacks the miR172 binding site (as for the *RcAP2L*^*∆172*^), its expression is maintained in the centre of the meristem, which leads to continuous downregulation of *AG* expression^30,44^. Such downregulation of *AG* expression results into the formation of flowers with an increased number of petals or stamens and a loss of floral determinacy^7,44^, a phenotype resembling that of *ag* loss-of-function mutant^45^. Similarly, *AG* expression is reduced when a miR172-resistant *TOE3* is expressed in *Arabidopsis* flowers^29^, indicating that other members of the euAP2 family can also have antagonistic role on the expression of *AG*.

In a recent study, Han *et al*.^46^, reported that the down-regulation of the rose *AP2* orthologue leads to the reduction of petal number^46^. However, the *RcAP2* gene (corresponding to *RcHm2g0106221*^27^) studied by Han *et al*.^46^, is located on chromosome 2 and not on chromosome 3, where the interval containing the double flower mutation lies.

In double flower roses, the *RcAP2L*^*∆172*^ truncated allele lost its miR172 binding site but still contained both AP2 DNA binding domains and EAR domains (Ethylene-responsive element binding factor-associated amphiphilic repression), thus consistent with an *RcAP2L* gain of function hypothesis and the dominant character of the *Df* gene. Indeed, *Arabidopsis* AP2 is known to interact with TOPLESS via its EAR domain to recruit the histone deacetylase HDA19 to its DNA binding sites including the *AG* second intron^47^. It has been demonstrated that a fusion between the AP2 DNA binding domains and TOPLESS, with the addition of an artificial miR172 binding site, is sufficient to complement the *Arabidopsis ap2* phenotype, indicating that the main floral function of AP2 is established via its TOPLESS interaction and recruitment to DNA target sites. Further investigation also revealed that TOE1, TOE2 and TOE3 interact with TOPLESS^48^, indicating a potentially conserved mechanism among the whole euAP2 family. Therefore, it is likely that in roses, the expression of the miR172 resistant allele (*RcAP2L*^*∆172*^) is responsible for the observed restricted expression of *RcAG* toward the centre of the flower, which in turn leads to the formation of flowers with an increased number of petals.

**Figure 4:**
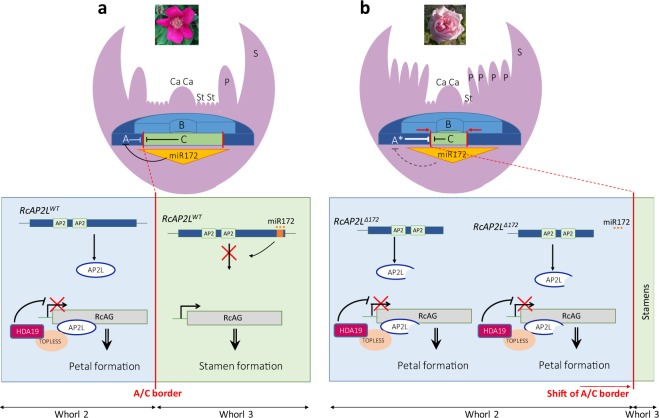
Model showing how a miR172-resistant euAP2 could lead to double flower formation. (**a**) In wild-type flowers, euAP2 are expressed in the first and second whorls where they can inhibit *RcAG* expression likely by recruiting cofactors and histone modifiers, such as *TOPLESS* and *HDA19*. Sepals and petals are consequently formed. In the 3^rd^ whorl, miR172 is expressed and inhibits euAP2 proteins production, releasing the inhibition of *RcAG*. *RcAG* will then determine stamens and carpels identity and formation. (**b**) In double flowers, the truncated version *RcAP2L*^*∆172*^ mRNA (following TE insertion) is insensitive to miR172 inhibition. *RcAP2L*^*∆172*^ expression is maintained in the meristem and down-regulates *RcAG* expression. This leads to the sliding of the A/C border toward the centre of the meristem and the formation of more petals, producing the so-called double-flowers. The wild-type *RcAP2L* (not shown on the Figure) is degraded. Ca, carpels; St, stamens; P, petals; S, sepals. A, A-class genes (*RcAP1*, *euAP2*); A*, A-class genes including a miR172-resistant euAP2 such as *RcAP2L*^*∆172*^; B, B-class genes; C, C-class gene (*RcAG*).

Our data, taken together with published data in *Arabidopsis* and other plants, suggest the following model. In simple flower roses, RcAP2L and RcAP2 proteins are produced only in the first two whorls, where they can inhibit *RcAG* transcription. This induces sepal and petal organ identity determination and development in whorls 1 and 2, respectively. In the third and fourth whorls, the accumulation of miR172 interferes with euAP2 mRNA accumulation, which in turn results in the expression of *RcAG* that mediates stamen and carpel organ identity determination and development (Fig. 4). However, in a double flower such as ‘Old Blush’, the presence of the miR172 insensitive variant *RcAP2L*^*∆172*^ leads to a prolonged accumulation of *RcAP2L* protein toward the centre of the meristem and consequently prolonged downregulation of *RcAG* expression (Fig. 4). This causes a restriction of *RcAG* expression toward the centre of the flower, as described previously^3^. As a consequence, the homeotic conversion of stamens into petals leads to the formation of a double flower.

Our model is supported by previous work on kiwifruit, where a downregulation of miR172 and an up-regulation of *AP2* were observed in flower buds from the “Pukekohe dwarf” kiwifruit double flower cultivars, but not in the “Hayward” and “Chieftain” simple flower cultivars^49^.

Our data taken together with that in the literature, strengthen the conclusion that misregulation of the miR172/*AP2* loop is likely the cause of the double flower phenotype in many species. To address more in deepth the molecular mechanisms that link the miR172 insensitive allele of *RcAP2L* to the double flower formation, a futur experiment would be to overexpress it in simple flower roses.

The fact that many double flower roses still develop carpels suggests that the accumulation of the miR172 insensitive variant *RcAP2L*^*∆172*^ affects *RcAG* expression only in the third whorl, but not in the fourth whorl. This indicates that the rose may have evolved differently from *Arabidopsis* where miR172 insensitive variant of *AP2* affect both whorls 3 and 4. It will be interesting to address the molecular mechanisms of such difference and whether such mechanism is applicable to other species with double flowers.

## Material and Methods

### Plant material

Double flower rose cultivars *Rosa chinensis* ‘Old Blush’, *R. odorata* ‘Hume’s Blush’, *R. x hybrida* ‘La France’, *R. x hybrida* ‘Rouge Meilland’, *R. x hybrida* ‘Bébé Fleuri’, *R. x hybrida* ‘Bengale d’Automne’, *R. x hybrida* ‘Cramoisi Supérieur’, *R. x hybrida* ‘Comtesse de Cayla’, *R. x hybrida* ‘Ducher’, *R. x hybrida* ‘General Shablikine’, *R. x hybrida* ‘Blush Noisette’, *R. x hybrida* ‘Herodiade’ and *R. x hybrida* ‘Louise d’Arzens’, and simple flower cultivars *R. chinensis* ‘Spontanea’, *R. chinensis* ‘Sanguinea’, *R. chinensis* ‘Mutabilis’, *R. wichurana*, *R. gigantea* and *R. moschata* were field grown at the Lyon-Botanical-Garden and/or in environmentally controlled greenhouse conditions at the Ecole Normale Supérieure of Lyon with 16 h/8 h day/night periods and 25 °C/18 °C day/night temperatures.

### Staging rose flower development

Flower development stages were distinguished and dissected under a binocular microscope as previously defined by Dubois *et al*.^14^. Stages 1 to 3 correspond to development stages when sepal, petal and stamen primordia arise, respectively. During stage 4, carpels are produced in the centre of the meristem, which will then sink below at stage 5.

### DNA extraction and genotyping PCR

Young leaves or axillary buds were collected and ground in PVP and homogenization buffer (Tris pH8 15 mM, EDTA 2 mM, NaCl 20 mM, KCl 20 mM, β-mercaptoethanol 0,1%, Triton 0,5%). DNA extraction was performed using the DNeasy kit (Qiagen).

DNA fragments were amplified using the GoTaq Polymerase Chain Reaction according to the manufacturer’s recommendation (Promega). An initial denaturing step was carried at 95 °C for 5 min. Fifteen cycles of touch-down PCR were then performed 95 °C for 30 s, 65 °C (with a decrease of 1 °C per cycle) for 30 s, 72 °C for 1 min 30 s. This was followed by 30 cycles of standard PCR with the following cycle 95 °C for 30 s, 50 °C for 30s and 72 °C for 1 min 30 s. A final elongation step was performed for 10 min at 72 °C.

### RNA purification and cDNA sequencing

Total RNA was prepared from floral meristems at different developmental stages (1 to 3) using the Spectrum plant total RNA kit (Sigma) and TURBO DNA-free^TM^ AM 1907 (Ambion), mainly as previously described^3^. Contaminating DNA was removed using the DNA-free^TM^ kit following the manufacturer’s recommendations (Ambion). One microgram of total RNA was then used in a reverse transcription assay. cDNAs were PCR amplified, cloned and sequenced using primers designed to specifically target *RcAP2L* or *RcAP2L*^*∆172*^ (Supplementary Table 3).

### Characterisation of the double flower interval

The high quality rose genome from *Rosa chinensis* ‘Old Blush’ was recently published^27^ in the form of two complementary assemblies. The first one was obtained from PacBio long read sequencing using a homozygous rose material derived from the heterozygous *Rosa chinensis* ‘Old Blush’^27,28^ and consists of 7 assembled pseudomolecules representing a haplotype of the rose genome. The genome of the heterozygous *Rosa chinensis* ‘Old Blush’ (Illumina sequencing) consists of 15,937 scaffolds and provides access to the two haplotypes of the genome.

Flanking markers of double flower interval^31^ were mapped on the *Rosa chinensis* homozygous reference genome^27^ using the following parameters: evalue < 10^−6^, lengthHSP > 40, percentage identity >97%. Markers that had unique match were kept and used to define the corresponding physical region on the rose genome sequence. Genes within this interval were analyzed using Blast and Pfam web interface^50,51^.

Analysis of the presence of the TE element in *RcAP2L* was also performed using the available genome sequences of rose cultivars^27^. Single reads from resequenced genomes of the different rose cultivars^27^ were trimmed using cutadapt^52^ and custom Perl scripts. They were cut to an homogeneous length of 100 bp and aligned on the reference rose genome using bwa software^53^ allowing up to two mismatches on the whole length of the read (end-to-end alignment). Reads overlapping genomic positions of interest over at least 15 bp on each side were counted. Read counts were normalized on the library size for each genotype. psRNATarget webserver interface was used to detect miR targets^54^.

### Haplotype identification and comparison

Sequence analysis was performed using the high-quality genome assembly of homozygous *R. chinensis* ‘Old Blush’^27^. The two distinct haplotypes within the double flower interval were retrieved from the heterozygous genome assembly^27^. Blastn^55^ and gene synteny were used to confirm alleles sequences. The water program from Emboss suite^56^ was used to obtain optimal end-to-end alignments between allelic regions and identify polymorphisms.

### Gene sequence analysis

Gene models were recovered from the rose reference genome sequence annotation^27^. Splicing site predictions and untranslated region (UTR) boundaries were manually adjusted based on cloned cDNA sequences and RNA-seq data. Putative functions for genes flanking *RcAP2L* were inferred from *Arabidopsis* best blast hit. Unknown protein domains were identified using InterProScan software version 5.27.-66.0^57^, Pfam database version 31.0^50^ and manual annotation. miR172 putative binding sites were predicted using a local instance of WMD3 software (Ossowski Stephan, Fitz Joffrey, Schwab Rebecca, Riester Markus and Weigel Detlef, personal communication).

### Transposable element annotation

To identify repeated regions, the genomic sequence of *RcAP2L* neighbourhood was cut into 47 bp overlapping *k*-mers, and the number of occurrences of each *k*-mer was counted in the 375 Gb-dataset of genomic reads used to assemble the *Rosa chinensis* heterozygous rose genome sequence^27^. These occurrence counts were plotted along the sequence (Fig. 1b) and compared to the mean occurrence counts for homozygous and heterozygous regions. Automatic transposable element (TE) annotations from the rose genome^27^ were used as a starting point, and manually curated.

The boundaries of the two long terminal repeats (LTRs) were accurately identified using a graphical dotplot program^58^ and LTR sequences were compared using bl2seq alignment^55^. Open reading frames were predicted in the TE internal region using Pfam software^50^ and protein domains were annotated by similarity search using DANTE (http://repeatexplorer.org/).

Using the LTR sequence as a Blastn query (parameters: M = 6 N = −7 Q = 8 R = 8; e-value ≤ 10^−20^, match length ≥600 bp) and the internal part sequence as tBlastx query (parameters: BLOSUM80 Q = 9 R = 3; e-value ≤ 10^−15^, overall coverage of query ≥800 bp), we looked for LTR pairs flanking a putative internal part, to detect complete copies of TEs from the same family. Using more stringent criteria (e-value ≤ 10^−80^ and match length ≥900 bp), we also identified solo-LTRs from the same family^59^.

### Expression analysis

Paired-end RNA-seq data from young flower buds at stage 1, 2 and 3 were previously described^27^.

Pairs of reads putatively originating from *RcAP2L*^*WT*^ and *RcAP2L*^*∆172*^ alleles were selected using Tophat version 2.1.1^60^ with relaxed parameters (“–read-realign-edit-dist 0–b2-very-sensitive –max-intron-length 25000” and insert size and insert size SD estimated beforehand on the whole predicted transcriptome for each library). These read pairs were remapped on the whole genome with Tophat allowing up to 5 multimatches and secondary alignments. Based on the number of matches and the alignment scores, read pairs were sorted into four categories: (i) specific to *RcAP2L*^*WT*^, (ii) specific to *RcAP2L*^*∆172*^, (iii) coming indiscriminately from *RcAP2L*^*WT*^ or *RcAP2L*^*∆172*^, and (iv) coming indiscriminately from *RcAP2L*^*WT*^, *RcAP2L*^*∆172*^ or other loci in the genome (hereafter called non-specific read pairs). Read pairs that Tophat could match on the extracted genomic sequences of *RcAP2L* alleles, but not on the whole genome, were put in category (iv). This case is expected for reads originating from repeated sequences. The read pairs from each category were mapped on the predicted transcripts using Bowtie2 version 2.3.4.1^61^. Coverage at each position was computed using Samtools version 1.5^62^. Normalization was done using the library sizes (custom Perl scripts), before adding up the coverage values from all libraries. Reads from categories (iii) and (iv) were spread between the two alleles according to the ratio (i)/(ii), computed on a sliding window of width 241 bp. After ensuring that sequence polymorphism between *RcAP2L*^*WT*^ and *RcAP2L*^*∆172*^ transcripts was sufficient to estimate independently their expression level, we used Tophat version 2.1.1^60^ on the annotated *Rosa chinensis* heterozygous genome^27^, with corrected annotations for *RcAP2L* alleles, and we normalized read counts using DESeq. 2 version 1.2.0^63^.

### Phylogenetic analysis

EuAP2 family members were identified by using the *Arabidopsis thaliana* AP2 protein as a Blast query against rose and strawberry predicted proteins^27^. Sequences were aligned using ClustalW^64^ and BioEdit software^65^. Where applicable, gene annotation was corrected manually.

Neighbor-Joining tree based on the aligned AP2 DNA binding domains of the euAP2 members from *Rosa chinensis* (RcHm and RcHt)^27^, *Fragaria vesca* (Fv), *Petunia*^12^, *Solanum lycopersicum*, *Arabidopsis thaliana*, *Capsella rubella*, *Medicago truncatula*, *Vitis vinifera and Prunus persica*. Sequences were downloaded from the Phytozome website (https://phytozome.jgi.doe.gov/pz/portal.html). The aligned regions containing the two AP2 domains (Supplemental Data File 1) were selected for phylogenetic analysis. Neighbor-Joining tree was computed with Treecon software^66^ using the following parameters (1) Distance estimation options: Tajima and Nei^67^; Distance calculations; insertions and deletion not taken into account; Alignment positions: all; Bootstrap analysis: yes, 2000 samples. (2) Infer tree topology options: Neighbor-joining; Bootstrap analysis: yes. (3) Root unrooted trees options: outgroup option: single sequence (forced); bootstrap analysis: yes. Tree was rooted using the *Arabidopsis* ANT protein.

## Electronic supplementary material

Supplementary Information
