# Supplementary material for: A miR172 target-deficient AP2-like gene correlates with the double flower phenotype in roses
Source: Sci Rep. 2018 Aug 27;8:12912. doi: 10.1038/s41598-018-30918-4 (PMC6110776; doi:10.1038/s41598-018-30918-4)
Supplement: Supplementary file 1 — Supplementary Information [file 41598_2018_30918_MOESM1_ESM.pdf]

Supplementary Information

A miR172 target-deficient AP2-like gene correlates with the double flower phenotype in roses

Léa FRANÇOIS[1], Marion VERDENAUD[1,2], Xiaopeng FU [1,3], Darcy RULEMAN [1], Annick DUBOIS[1], Michiel VANDENBUSSCHE[1], Abdelhafid BENDAHMANE[2], Olivier RAYMOND[1], Jérémy JUST[1], Mohammed BENDAHMANE[1*]

[1]Laboratoire Reproduction et Développement des Plantes, Univ Lyon, ENS de Lyon, UCB Lyon 1, CNRS, INRA, F-69364 Lyon, France.

[2]Institute of Plant Sciences Paris-Saclay IPS2, CNRS, INRA, University Paris-Sud, University of Evry, University Paris- Diderot, Sorbonne Paris-Cite, University of Paris-Saclay, F-91405 Orsay, France.

[3]Key Lab of Horticultural Plant Biology, College of Horticulture & Forestry Sciences, Huazhong Agricultural University, Wuhan, China.

[*]Corresponding author: Mohammed BENDAHMANE,
E-mail: mohammed.bendahmane@ens-lyon

Supplementary Table 1

1	RcHm3g0467401	soluble inorganic pyrophosphatase 4
2	RcHm3g0467411	Unknown function
3	RcHm3g0467421	ras-related RABE1a
4	RcHm3g0467431	WAT1-related At2g37460-like
5	RcHm3g0467441	PREDICTED: uncharacterized protein LOC101295827 isoform X1
6	RcHm3g0467461	type I inositol 1,4,5-trisphosphate 5-phosphatase CVP2
7	RcHm3g0467471	Unknown function
8	RcHm3g0467481	midasin isoform X2
9	RcHm3g0467491	Unknown function
10	RcHm3g0467501	zinc finger ZAT11-like
11	RcHm3g0467521	zinc finger ZAT11-like
12	RcHm3g0467531	zinc finger ZAT11-like
13	RcHm3g0467541	zinc finger ZAT11-like
14	RcHm3g0467551	125 kDa kinesin-related
15	RcHm3g0467561	WUSCHEL ortholog
16	RcHm3g0467571	diaminopimelate chloroplastic-like
17	RcHm3g0467581	F-box At2g39490-like
18	RcHm3g0467591	Unknown function
19	RcHm3g0467601	PREDICTED: uncharacterized protein LOC105352633
20	RcHm3g0467611	pentatricopeptide repeat-containing mitochondrial-like
21	RcHm3g0467621	serine threonine- kinase AFC1
22	RcHm3g0467631	heat shock cognate 70 kDa -like
23	RcHm3g0467641	heat shock cognate 70 kDa -like
24	RcHm3g0467651	heat shock cognate 70 kDa -like
25	RcHm3g0467661	heat shock cognate 70 kDa -like
26	RcHm3g0467671	heat shock cognate 70 kDa -like
27	RcHm3g0467681	Argininosuccinate lyase
28	RcHm3g0467691	WUSCHEL
29	RcHm3g0467701	SLOW GREEN chloroplastic-like
30	RcHm3g0467721	probable GPI-anchored adhesin PGA55
31	RcHm3g0467731	disease resistance RGA1 isoform X1
32	RcHm3g0467741	Hydroxyproline-rich glycofamily
33	RcHm3g0467751	chloroplast-targeted copper
34	RcHm3g0467761	UDP-glucuronic acid decarboxylase 1
35	RcHm3g0467771	Unknown function
36	RcHm3g0467781	Unknown function
37	RcHm3g0467791	DNA RNA polymerases superfamily
38	RcHm3g0467801	Unknown function
39	RcHm3g0467811	Unknown function
40	RcHm3g0467831	exocyst complex component EXO70A1-like
41	RcHm3g0467841	WUSCHEL
42	RcHm3g0467851	exocyst complex component EXO70A1-like
43	RcHm3g0467861	probable pectate lyase 4
44	RcHm3g0467881	probable caffeoyl- O-methyltransferase At4g26220
45	RcHm3g0467891	ankyrin repeat-containing At3g12360-like
46	RcHm3g0467901	ankyrin repeat-containing At5g02620-like
47	RcHm3g0467911	probable membrane-associated kinase regulator 4
48	RcHm3g0467921	Unknown function
49	RcHm3g0467931	myosin-11 isoform X1
50	RcHm3g0467941	phosphatidate phosphatase PAH1-like
51	RcHm3g0467951	GPR107-like
52	RcHm3g0467961	PREDICTED: uncharacterized protein LOC101314581
53	RcHm3g0467971	alpha partial
54	RcHm3g0467981	calcium-transporting ATPase endoplasmic reticulum-type-like
55	RcHm3g0467991	ABC transporter G family member 20-like
56	RcHm3g0468001	serine arginine-rich splicing factor RS2Z32 isoform X1
57	RcHm3g0468011	Retinoic acid-induced 3
58	RcHm3g0468021	PREDICTED: uncharacterized protein LOC101291256
59	RcHm3g0468031	pathogenesis-related 1A-like
60	RcHm3g0468041	Unknown function
61	RcHm3g0468051	ALUMINUM SENSITIVE 3-like
62	RcHm3g0468061	PREDICTED: uncharacterized protein LOC101292034 isoform X1
63	RcHm3g0468071	pentatricopeptide repeat-containing At2g37320
64	RcHm3g0468081	dirigent 17-like
65	RcHm3g0468091	pentatricopeptide repeat-containing At2g37310
66	RcHm3g0468101	E3 ubiquitin- ligase amyloplastic
67	RcHm3g0468111	zinc finger CCHC domain-containing 10
68	RcHm3g0468121	probable phosphatase 2C 38
69	RcHm3g0468131	MODIFIER OF SNC1 11
70	RcHm3g0468141	thylakoid membrane chloroplastic
71	RcHm3g0468151	glutathione S-transferase L3-like
72	RcHm3g0468161	glutathione S-transferase L3-like
73	RcHm3g0468171	F-box CPR30-like
74	RcHm3g0468181	Retrovirus-related Pol poly from transposon TNT 1-94
75	RcHm3g0468191	F-box CPR30-like
76	RcHm3g0468201	TBC1 domain family member 8B-like isoform X1
77	RcHm3g0468211	serine threonine- kinase PBS1
78	RcHm3g0468221	WRKY transcription factor 44
79	RcHm3g0468231	pentatricopeptide repeat-containing mitochondrial
80	RcHm3g0468241	two-component response regulator-like APRR7
81	RcHm3g0468251	adenylate kinase chloroplastic-like
82	RcHm3g0468261	thioredoxin chloroplastic
83	RcHm3g0468271	Unknown function
84	RcHm3g0468281	UDP-galactose UDP-glucose transporter 2-like
85	RcHm3g0468291	disease resistance RGA1
86	RcHm3g0468301	Unknown function
87	RcHm3g0468321	disease resistance RGA1
88	RcHm3g0468331	Unknown function
89	RcHm3g0468341	disease resistance RGA1
90	RcHm3g0468351	pentatricopeptide repeat-containing chloroplastic
91	RcHm3g0468371	eukaryotic translation initiation factor 2 subunit alpha-like
92	RcHm3g0468381	Unknown function
93	RcHm3g0468391	disease resistance RGA1 isoform X1
94	RcHm3g0468401	Unknown function
95	RcHm3g0468411	disease resistance RGA1
96	RcHm3g0468421	pentatricopeptide repeat-containing chloroplastic
97	RcHm3g0468431	zinc finger CCCH domain-containing 11
98	RcHm3g0468441	Unknown function
99	RcHm3g0468451	2,3-bisphosphoglycerate-independent phosphoglycerate mutase
100	RcHm3g0468461	REVEILLE 8 isoform X1
101	RcHm3g0468471	60S ribosomal L4
102	**RcHm3g0468481**	**floral homeotic APETALA 2-like isoform X1**
103	**RcHm3g0468491**	**floral homeotic APETALA 2-like isoform X1**
104	RcHm3g0468501	pentatricopeptide repeat-containing mitochondrial-like
105	RcHm3g0468521	Unknown function
106	RcHm3g0468531	PREDICTED: uncharacterized protein LOC105353349, partial
107	RcHm3g0468541	PREDICTED: uncharacterized protein LOC101305581
108	RcHm3g0468551	L-ascorbate peroxidase cytosolic
109	RcHm3g0468561	probable LRR receptor-like serine threonine- kinase At1g67720
110	RcHm3g0468571	disease resistance RGA1 isoform X1
111	RcHm3g0468591	disease resistance RGA1
112	RcHm3g0468601	Fasciclin domain-containing isoform 1
113	RcHm3g0468611	inosine-5 -monophosphate dehydrogenase 2-like
114	RcHm3g0468621	pumilio homolog 12
115	RcHm3g0468641	Unknown function
116	RcHm3g0468651	hypothetical protein PRUPE_ppa016183mg
117	RcHm3g0468661	nuclear transcription factor Y subunit B-10-like
118	RcHm3g0468671	E3 ubiquitin- ligase UPL4
119	RcHm3g0468681	proteasome subunit beta type-7-A
120	RcHm3g0468691	Unknown function
121	RcHm3g0468701	pentatricopeptide repeat-containing chloroplastic
122	RcHm3g0468711	probable DNA helicase MCM8 isoform X1
123	RcHm3g0468721	Tudor PWWP MBT superfamily isoform 1
124	RcHm3g0468731	ribonuclease H At1g65750
125	RcHm3g0468741	non-ltr retroelement reverse transcriptase
126	RcHm3g0468751	PREDICTED: uncharacterized protein LOC103334450
127	RcHm3g0468761	PREDICTED: uncharacterized protein LOC105352505
128	RcHm3g0468771	PREDICTED: uncharacterized protein LOC103422832
129	RcHm3g0468781	F-box LRR-repeat 4-like
130	RcHm3g0468791	Tudor PWWP MBT superfamily isoform 1
131	RcHm3g0468801	probable GPI-anchored adhesin PGA55
132	RcHm3g0468811	cyclic nucleotide-gated ion channel 1-like
133	RcHm3g0468821	cyclic nucleotide-gated ion channel 1-like
134	RcHm3g0468831	Unknown function
135	RcHm3g0468851	interactor of constitutive active ROPs chloroplastic-like isoform X1
136	RcHm3g0468861	hydrophobic RCI2A
137	RcHm3g0468871	Zinc finger -
138	RcHm3g0468881	ribonuclease P subunit p29
139	RcHm3g0468891	hydrolase family
140	RcHm3g0468901	mitochondrial import inner membrane translocase subunit TIM14-1
141	RcHm3g0468911	CWC15 homolog
142	RcHm3g0468921	IQ-DOMAIN 1
143	RcHm3g0468931	probable beta-1,4-xylosyltransferase IRX9
144	RcHm3g0468941	pentatricopeptide repeat-containing mitochondrial
145	RcHm3g0468951	DEAD-box ATP-dependent RNA helicase 57
146	RcHm3g0468961	importin subunit alpha-9
147	RcHm3g0468971	lipid phosphate phosphatase chloroplastic
148	RcHm3g0468981	probable disease resistance At4g27220
149	RcHm3g0468991	zinc finger BED domain-containing RICESLEEPER 2-like
150	RcHm3g0469001	POLAR LOCALIZATION DURING ASYMMETRIC DIVISION AND REDISTRIBUTION
151	RcHm3g0469011	chromo domain-containing cec-1
152	RcHm3g0469021	defensin Tk-AMP-D1-like
153	RcHm3g0469031	PREDICTED: uncharacterized protein LOC101304113
154	RcHm3g0469041	Unknown function
155	RcHm3g0469051	cell number regulator 8
156	RcHm3g0469061	KRR1 small subunit processome component homolog
157	RcHm3g0469071	Poly(A) polymerase
158	RcHm3g0469081	KRR1 small subunit processome component homolog
159	RcHm3g0469101	mitochondrial phosphate carrier mitochondrial-like
160	RcHm3g0469111	DNA-binding S1FA-like
161	RcHm3g0469121	L-type lectin-domain containing receptor kinase
162	RcHm3g0469131	Unknown function
163	RcHm3g0469141	peroxidase 21
164	RcHm3g0469151	transcription initiation factor TFIID subunit 7-like
165	RcHm3g0469161	hypothetical protein PRUPE_ppa017061mg
166	RcHm3g0469171	MLO 3
167	RcHm3g0469191	MLO 6
168	RcHm3g0469201	ribonuclease H At1g65750
169	RcHm3g0469211	aspartic ase nepenthesin-1-like
170	RcHm3g0469221	probable E3 ubiquitin- ligase ARI7
171	RcHm3g0469231	Unknown function
172	RcHm3g0469241	Unknown function
173	RcHm3g0469251	Unknown function
174	RcHm3g0469261	Unknown function
175	RcHm3g0469271	Unknown function
176	RcHm3g0469281	Unknown function
177	RcHm3g0469291	polyubiquitin 3
178	RcHm3g0469301	DNA-directed RNA polymerase III subunit rpc1
179	RcHm3g0469311	Unknown function
180	RcHm3g0469321	DNA-directed RNA polymerase III subunit rpc1
181	RcHm3g0469331	polyubiquitin isoform X1
182	RcHm3g0469341	E3 ubiquitin- ligase RLIM isoform X1
183	RcHm3g0469351	YLS9-like
184	RcHm3g0469361	syntaxin-71-like
185	RcHm3g0469371	NAC domain-containing 29
186	RcHm3g0469381	zinc finger JACKDAW-like
187	RcHm3g0469391	dnaJ P58IPK homolog
188	RcHm3g0469401	fasciclin-like arabinogalactan 11
189	RcHm3g0469411	WD repeat-containing 20-like
190	RcHm3g0469421	Binding isoform 1
191	RcHm3g0469431	WUSCHEL ortholog
192	RcHm3g0469441	Unknown function
193	RcHm3g0469451	PREDICTED: uncharacterized protein LOC101308990
194	RcHm3g0469461	probable E3 ubiquitin- ligase LOG2
195	RcHm3g0469471	serine threonine- kinase
196	RcHm3g0469481	serine threonine- kinase CCR1
197	RcHm3g0469501	SEC14 cytosolic factor-like
198	RcHm3g0469511	serine threonine- kinase ATM-like
199	RcHm3g0469521	V-type proton ATPase subunit G1-like
200	RcHm3g0469531	aquaporin PIP2-1-like
201	RcHm3g0469541	aquaporin PIP2-1-like
202	RcHm3g0469551	40S ribosomal S16
203	RcHm3g0469561	hypothetical protein B456_009G418000
204	RcHm3g0469571	hypothetical protein PHAVU_007G220400g
205	RcHm3g0469581	60S ribosomal L12
206	RcHm3g0469591	ubiquitin carboxyl-terminal hydrolase 34-like
207	RcHm3g0469601	PREDICTED: uncharacterized protein LOC101312560
208	RcHm3g0469611	TIC 20- chloroplastic-like
209	RcHm3g0469621	wall-associated receptor kinase-like 1
210	RcHm3g0469631	wall-associated receptor kinase-like 1
211	RcHm3g0469641	CASP 5A1
212	RcHm3g0469651	ubiquitin 5
213	RcHm3g0469661	auxin response factor 18-like isoform X2
214	RcHm3g0469671	PREDICTED: uncharacterized protein LOC101314108
215	RcHm3g0469681	Unknown function
216	RcHm3g0469691	cytochrome P450 87A3
217	RcHm3g0469701	Unknown function
218	RcHm3g0469711	PREDICTED: uncharacterized protein LOC104894399
219	RcHm3g0469721	Unknown function
220	RcHm3g0469731	BTB POZ domain-containing At5g03250
221	RcHm3g0469741	cytokinin riboside 5 -monophosphate phosphoribohydrolase LOG3
222	RcHm3g0469751	Unknown function
223	RcHm3g0469761	Unknown function
224	RcHm3g0469771	29 kDa ribonucleo chloroplastic
225	RcHm3g0469781	synaptotagmin-3-like
226	RcHm3g0469791	ethylene-insensitive 2
227	RcHm3g0469801	hypothetical protein L484_024375
228	RcHm3g0469811	Unknown function
229	RcHm3g0469821	Homeodomain-like superfamily
230	RcHm3g0469831	anaphase-promoting complex subunit 6
231	RcHm3g0469841	40S ribosomal S15a-1
232	RcHm3g0469851	Wuschel
233	RcHm3g0469861	phenylalanine ammonia lyase
234	RcHm3g0469871	casein kinase I-like
235	RcHm3g0469881	AUGMIN subunit 7-like
236	RcHm3g0469891	orf120 gene product (mitochondrion)
237	RcHm3g0469901	isocitrate dehydrogenase [NAD] catalytic subunit mitochondrial
238	RcHm3g0469911	adenosine kinase 2
239	RcHm3g0469921	adenosine kinase 2
240	RcHm3g0469941	PREDICTED: uncharacterized protein LOC101291848
241	RcHm3g0469951	indole-3-acetic acid-induced ARG7
242	RcHm3g0469961	Unknown function
243	RcHm3g0469971	Telomere repeat-binding 4
244	RcHm3g0469981	translin-like
245	RcHm3g0469991	white-brown complex homolog 30
246	RcHm3g0470001	probable receptor kinase At5g47070
247	RcHm3g0470011	OTU domain-containing DDB_G0284757
248	RcHm3g0470021	transcription factor TCP11
249	RcHm3g0470031	RNA polymerase sigma factor chloroplastic isoform X1
250	RcHm3g0470041	pentatricopeptide repeat-containing At2g32630
251	RcHm3g0470051	DVL family
252	RcHm3g0470061	cell division cycle 48 homolog
253	RcHm3g0470071	UDP-glycosyltransferase 86A1-like
254	RcHm3g0470081	WUSCHEL
255	RcHm3g0470091	aspartate chloroplastic
256	RcHm3g0470101	UDP-glycosyltransferase 86A1-like
257	RcHm3g0470111	UDP-glycosyltransferase 86A1-like
258	RcHm3g0470121	TSL-kinase interacting 1 isoform X1
259	RcHm3g0470131	phosphoglycerate cytosolic-like isoform X1
260	RcHm3g0470141	membrane magnesium transporter-like
261	RcHm3g0470151	transcription initiation factor TFIID subunit 8-like
262	RcHm3g0470161	Actin-related T1
263	RcHm3g0470171	thylakoid lumenal kDa chloroplastic
264	RcHm3g0470181	calcium-dependent kinase 10-like
265	RcHm3g0470191	Retrovirus-related Pol poly from transposon TNT 1-94
266	RcHm3g0470201	Cyclin family isoform 1
267	RcHm3g0470211	CLAVATA 3
268	RcHm3g0470221	SAUR-like auxin-responsive
269	RcHm3g0470231	SAUR-like auxin-responsive
270	RcHm3g0470251	Acylphosphatase family isoform 1
271	RcHm3g0470261	Unknown function
272	RcHm3g0470271	axoneme-associated mst101(2)-like isoform X1
273	RcHm3g0470281	Unknown function
274	RcHm3g0470291	histidine--tRNA cytoplasmic
275	RcHm3g0470301	zinc finger 593
276	RcHm3g0470311	ABC transporter B family member 1
277	RcHm3g0470321	Isoleucine N-monooxygenase 2
278	RcHm3g0470331	Wuschel
279	RcHm3g0470341	phenylalanine N-monooxygenase-like
280	RcHm3g0470361	PREDICTED: uncharacterized protein LOC101297757
281	RcHm3g0470371	Unknown function
282	RcHm3g0470381	transcription factor RAX2
283	RcHm3g0470391	probable GPI-anchored adhesin PGA55 isoform X3
284	RcHm3g0470401	GATA zinc finger domain-containing 10
285	RcHm3g0470411	jacalin-related lectin 3-like
286	RcHm3g0470421	S-adenosylmethionine synthase 3
287	RcHm3g0470431	thioredoxin-like 3-3
288	RcHm3g0470441	FAD synthase isoform X1
289	RcHm3g0470451	Bifunctional aas
290	RcHm3g0470461	probable xyloglucan endotransglucosylase hydrolase 32
291	RcHm3g0470481	E3 ubiquitin- ligase RFWD3
292	RcHm3g0470491	serine threonine- kinase
293	RcHm3g0470501	probable leucine-rich repeat receptor kinase At5g63930
294	RcHm3g0470511	dual specificity phosphatase Cdc25
295	RcHm3g0470531	Polypeptide N-acetylgalactosaminyltransferase 35A
296	RcHm3g0470541	ACT domain-containing ACR10-like
297	RcHm3g0470551	Unknown function
298	RcHm3g0470561	Unknown function
299	RcHm3g0470571	WUSCHEL
300	RcHm3g0470581	heparan-alpha-glucosaminide N-acetyltransferase-like
301	RcHm3g0470591	NUCLEAR FUSION DEFECTIVE 4-like
302	RcHm3g0470601	serine threonine phosphatase 2A 57 kDa regulatory subunit B beta isoform
303	RcHm3g0470611	PREDICTED: uncharacterized protein LOC101301891
304	RcHm3g0470621	myb-related 305 isoform X1
305	RcHm3g0470631	probable pectate lyase 12
306	RcHm3g0470641	alpha carbonic anhydrase 7-like
307	RcHm3g0470651	AT-rich interactive domain-containing 1-like isoform X4
308	RcHm3g0470661	bifunctional monodehydroascorbate reductase and carbonic anhydrase nectarin-3-
309	RcHm3g0470681	bifunctional monodehydroascorbate reductase and carbonic anhydrase nectarin-3-
310	RcHm3g0470691	RING finger and CHY zinc finger domain-containing 1-like
311	RcHm3g0470701	aquaporin TIP1-1
312	RcHm3g0470721	UDP-sugar pyrophosphorylase
313	RcHm3g0470731	Sec14p-like phosphatidylinositol transfer family isoform 1
314	RcHm3g0470741	PREDICTED: uncharacterized protein LOC101309548
315	RcHm3g0470751	homeobox-leucine zipper HDG2-like isoform X1
316	RcHm3g0470761	Unknown function
317	RcHm3g0470771	PREDICTED: uncharacterized protein LOC101303440
318	RcHm3g0470781	Unknown function
319	RcHm3g0470791	Unknown function
320	RcHm3g0470801	NADH-plastoquinone oxidoreductase subunit partial (chloroplast)
321	RcHm3g0470811	NADH-plastoquinone oxidoreductase subunit 7
322	RcHm3g0470821	ribosomal S15 (chloroplast)
323	RcHm3g0470831	zinc finger ZAT5-like
324	RcHm3g0470841	Ankyrin repeat family isoform 1
325	RcHm3g0470851	bifunctional monodehydroascorbate reductase and carbonic anhydrase nectarin-3-
326	RcHm3g0470861	pentatricopeptide repeat-containing At2g33680-like
327	RcHm3g0470871	bifunctional monodehydroascorbate reductase and carbonic anhydrase nectarin-3-
328	RcHm3g0470881	pentatricopeptide repeat-containing At2g33680-like
329	RcHm3g0470891	ras-related RABC2a-like
330	RcHm3g0470901	splicing factor 3A subunit 2-like
331	RcHm3g0470911	phosphatidylinositol 4-phosphate 5-kinase 9
332	RcHm3g0470921	WD repeat-containing 3
333	RcHm3g0470931	expansin-A9-like
334	RcHm3g0470941	expansin-A9-like
335	RcHm3g0470951	probable LRR receptor-like serine threonine- kinase At3g47570
336	RcHm3g0470961	expansin-A9-like
337	RcHm3g0470971	fluG
338	RcHm3g0470981	thaumatin precursor
339	RcHm3g0470991	two-pore potassium channel 5
340	RcHm3g0471001	SHOOT GRAVITROPISM 6 isoform X1
341	RcHm3g0471011	UDP-glycosyltransferase 73C1-like
342	RcHm3g0471031	2-aminoethanethiol dioxygenase
343	RcHm3g0471041	Unknown function
344	RcHm3g0471051	UDP-glycosyltransferase 73C1-like
345	RcHm3g0471061	Unknown function
346	RcHm3g0471071	UDP-glycosyltransferase 73C1-like
347	RcHm3g0471081	UDP-glycosyltransferase 73C1-like
348	RcHm3g0471091	UDP-glycosyltransferase 73C3-like
349	RcHm3g0471101	UDP-glycosyltransferase 73C3-like
350	RcHm3g0471111	UDP-Glycosyltransferase superfamily
351	RcHm3g0471121	UDP-Glycosyltransferase superfamily
352	RcHm3g0471131	UDP-glycosyltransferase 73D1
353	RcHm3g0471141	Unknown function
354	RcHm3g0471151	UDP-glycosyltransferase 73D1
355	RcHm3g0471171	probable pectinesterase 15
356	RcHm3g0471181	Chromodomain-helicase-DNA-binding 4
357	RcHm3g0471191	Unknown function
358	RcHm3g0471201	leucine-rich repeat-containing DDB_G0290503
359	RcHm3g0471211	caffeic acid 3-O-methyltransferase
360	RcHm3g0471221	acetylglutamate chloroplastic
361	RcHm3g0471231	carotene epsilon- chloroplastic
362	RcHm3g0471241	Unknown function
363	RcHm3g0471251	probable 2-oxoglutarate Fe(II)-dependent dioxygenase
364	RcHm3g0471261	uncharacterized permease
365	RcHm3g0471271	RING-H2 finger ATL52-like
366	RcHm3g0471281	PREDICTED: uncharacterized protein LOC101301510
367	RcHm3g0471291	vacuolar -sorting-associated 37 homolog 1
368	RcHm3g0471301	dnaJ homolog subfamily C member 28-like
369	RcHm3g0471311	DEAD-box ATP-dependent RNA helicase 38
370	RcHm3g0471321	DEAD-box ATP-dependent RNA helicase 38
371	RcHm3g0471331	NAC domain-containing 67-like
372	RcHm3g0471341	Unknown function
373	RcHm3g0471351	pistil-specific extensin
374	RcHm3g0471361	DEAD-box ATP-dependent RNA helicase 38
375	RcHm3g0471371	YLS9-like
376	RcHm3g0471381	PELOTA 1-like
377	RcHm3g0471391	DEAD-box ATP-dependent RNA helicase 38
378	RcHm3g0471401	aspartic ase 2
379	RcHm3g0471411	Unknown function
380	RcHm3g0471421	polyadenylate-binding 7
381	RcHm3g0471431	Unknown function
382	RcHm3g0471441	pectinesterase pectinesterase inhibitor PPE8B-like
383	RcHm3g0471451	cactin
384	RcHm3g0471461	wall-associated receptor kinase-like 20
385	RcHm3g0471471	peptidyl-prolyl cis-trans isomerase FKBP16- chloroplastic
386	RcHm3g0471481	chloroplastic
387	RcHm3g0471491	Unknown function
388	RcHm3g0471501	threonine dehydratase chloroplastic
389	RcHm3g0471511	GDSL esterase lipase At5g03820-like
390	RcHm3g0471521	GDSL esterase lipase At5g03820-like
391	RcHm3g0471531	ribonuclease H At1g65750
392	RcHm3g0471541	E3 ubiquitin- ligase UPL7 isoform X1
393	RcHm3g0471551	Unknown function
394	RcHm3g0471561	probable salt tolerance At1g78600
395	RcHm3g0471571	pentatricopeptide repeat-containing At5g03800
396	RcHm3g0471581	YLS9-like
397	RcHm3g0471591	pentatricopeptide repeat-containing At5g03800
398	RcHm3g0471601	cactin
399	RcHm3g0471611	RNA-dependent RNA polymerase 1-like
400	RcHm3g0471621	F-box LRR-repeat At3g26922-like
401	RcHm3g0471631	Unknown function
402	RcHm3g0471641	probable glycosyltransferase At5g03795
403	RcHm3g0471651	embryonic DC-8
404	RcHm3g0471661	Sulfite exporter family isoform 1
405	RcHm3g0471671	SRSF kinase 1
406	RcHm3g0471681	60S ribosomal L24-like
407	RcHm3g0471691	homeobox-leucine zipper ATHB-51
408	RcHm3g0471701	F-box PP2-A15
409	RcHm3g0471711	stress response NST1
410	RcHm3g0471721	pyruvate cytosolic isozyme-like
411	RcHm3g0471731	Uridylate kinase
412	RcHm3g0471741	probable leucine-rich repeat receptor kinase At1g68400
413	RcHm3g0471751	L-type lectin-domain containing receptor kinase -like
414	RcHm3g0471761	Unknown function
415	RcHm3g0471771	auxin-induced 15A-like
416	RcHm3g0471781	probable 3-deoxy-D-manno-octulosonic acid mitochondrial isoform X1
417	RcHm3g0471791	cytochrome P450 76A1-like
418	RcHm3g0471801	cytochrome P450 76A2-like
419	RcHm3g0471811	BOBBER 1-like
420	RcHm3g0471821	Unknown function
421	RcHm3g0471831	probable thylakoidal processing peptidase chloroplastic
422	RcHm3g0471841	Unknown function
423	RcHm3g0471851	F-box kelch-repeat At1g12870
424	RcHm3g0471861	PREDICTED: uncharacterized protein LOC101310039
425	RcHm3g0471871	AT-hook motif nuclear-localized 1-like
426	RcHm3g0471881	NLI interacting factor-like phosphatase
427	RcHm3g0471891	Unknown function
428	RcHm3g0471901	probable C-terminal domain small phosphatase
429	RcHm3g0471911	PREDICTED: uncharacterized protein LOC101308886
430	RcHm3g0471921	cullin-1
431	RcHm3g0471931	Unknown function
432	RcHm3g0471941	hypothetical protein DCAR_031676
433	RcHm3g0471951	Unknown function
434	RcHm3g0471961	glucomannan 4-beta-mannosyltransferase 9
435	RcHm3g0471971	pentatricopeptide repeat-containing mitochondrial
436	RcHm3g0471981	peroxiredoxin-2E- chloroplastic
437	RcHm3g0471991	Unknown function
438	RcHm3g0472001	enolase
439	RcHm3g0472021	splicing factor U2af small subunit B-like
440	RcHm3g0472031	Unknown function
441	RcHm3g0472041	histone deacetylase HDT1-like
442	RcHm3g0472051	Nucleotide-diphospho-sugar transferases superfamily
443	RcHm3g0472061	Gamma-tocopherol methyltransferase
444	RcHm3g0472071	Unknown function
445	RcHm3g0472081	delta(14)-sterol reductase
446	RcHm3g0472091	ROS1
447	RcHm3g0472101	serine threonine- kinase CTR1
448	RcHm3g0472111	Unknown function
449	RcHm3g0472131	heat stress transcription factor A-3
450	RcHm3g0472141	heat stress transcription factor A-3
451	RcHm3g0472151	ribosome production factor 2 homolog
452	RcHm3g0472161	probable GPI-anchored adhesin PGA55 isoform X2
453	RcHm3g0472171	phytosulfokines 6
454	RcHm3g0472181	Unknown function
455	RcHm3g0472201	PAN domain-containing At5g03700
456	RcHm3g0472211	PREDICTED: uncharacterized protein LOC101294969
457	RcHm3g0472221	phenylalanine N-monooxygenase-like
458	RcHm3g0472231	Isoleucine N-monooxygenase 2
459	RcHm3g0472241	Unknown function
460	RcHm3g0472251	fructose-bisphosphate aldolase cytoplasmic isozyme
461	RcHm3g0472261	fructose-bisphosphate aldolase cytoplasmic isozyme
462	RcHm3g0472271	Trihelix transcription factor GT-2
463	RcHm3g0472281	ethylene-responsive transcription factor ERF024
464	RcHm3g0472291	vesicle-fusing ATPase
465	RcHm3g0472301	NLI interacting factor-like phosphatase
466	RcHm3g0472311	Uncharacterized FCP1-like domain-containing
467	RcHm3g0472321	Uncharacterized protein TCM_022470
468	RcHm3g0472331	UPF0481 At3g47200-like
469	RcHm3g0472341	UPF0481 At3g47200-like
470	RcHm3g0472351	40S ribosomal S15a-1
471	RcHm3g0472361	dehydration-responsive element-binding 1B-like
472	RcHm3g0472371	histone-lysine N-methyltransferase SETD1B-like
473	RcHm3g0472381	probable DNA double-strand break repair Rad50 ATPase
474	RcHm3g0472391	Unknown function
475	RcHm3g0472401	probable ribose-5-phosphate isomerase 2
476	RcHm3g0472411	growth-regulating factor 3
477	RcHm3g0472421	starch branching enzyme I
478	RcHm3g0472431	calcineurin-like phosphoesterase
479	RcHm3g0472441	calcineurin-like phosphoesterase
480	RcHm3g0472451	pleiotropic drug resistance 2-like
481	RcHm3g0472461	BUD31 homolog 2
482	RcHm3g0472481	linoleate 13S-lipoxygenase 3- chloroplastic-like
483	RcHm3g0472491	BTB POZ domain-containing FBL11 isoform X1
484	RcHm3g0472501	BTB POZ domain-containing FBL11 isoform X1
485	RcHm3g0472511	KCBP-interacting isoform 1
486	RcHm3g0472521	monodehydroascorbate reductase
487	RcHm3g0472531	Unknown function
488	RcHm3g0472541	60S acidic ribosomal P2-2-like
489	RcHm3g0472551	Unknown function
490	RcHm3g0472561	UPF0481 At3g47200-like
491	RcHm3g0472571	Unknown function
492	RcHm3g0472581	pentatricopeptide repeat-containing At1g10270
493	RcHm3g0472601	DNA-directed RNA polymerase V subunit 5C
494	RcHm3g0472611	UPF0481 At3g47200-like
495	RcHm3g0472621	UPF0481 At3g47200-like
496	RcHm3g0472631	UPF0481 At3g47200-like
497	RcHm3g0472641	IQ calmodulin-binding motif family
498	RcHm3g0472651	histidine-containing phosphotransfer 1-like
499	RcHm3g0472661	Transcription factor HBP-1a
500	RcHm3g0472671	subtilisin-like protease
501	RcHm3g0472681	subtilisin-like protease
502	RcHm3g0472691	DNA binding isoform 1
503	RcHm3g0472701	DNA binding
504	RcHm3g0472711	probable LRR receptor-like serine threonine- kinase At4g36180
505	RcHm3g0472721	cytochrome P450 94C1-like
506	RcHm3g0472731	GDSL esterase lipase At5g03610-like
507	RcHm3g0472741	Unknown function
508	RcHm3g0472751	DNA topoisomerase 6 subunit B
509	RcHm3g0472761	Unknown function
510	RcHm3g0472771	PREDICTED: uncharacterized protein LOC101298622
511	RcHm3g0472781	PREDICTED: uncharacterized protein LOC105349503
512	RcHm3g0472791	ribonuclease H At1g65750
513	RcHm3g0472801	GDSL esterase lipase At5g03610-like
514	RcHm3g0472811	FK506-binding 5-like
515	RcHm3g0472821	PREDICTED: uncharacterized protein LOC103455563
516	RcHm3g0472831	GDSL esterase lipase At5g03610-like
517	RcHm3g0472841	ribonuclease H At1g65750
518	RcHm3g0472851	uridine nucleosidase 1
519	RcHm3g0472861	transcription initiation factor TFIID subunit 12
520	RcHm3g0472871	ethylene-responsive transcription factor At4g13040
521	RcHm3g0472881	solute carrier family 35 member F1-like
522	RcHm3g0472891	zinc finger A20 and AN1 domain-containing stress-associated 4
523	RcHm3g0472901	Unknown function
524	RcHm3g0472911	hypothetical protein GLYMA_08G175800
525	RcHm3g0472921	purple acid phosphatase 22-like
526	RcHm3g0472931	Unknown function
527	RcHm3g0472941	purple acid phosphatase 22-like
528	RcHm3g0472951	eukaryotic initiation factor 4A isoform X1
529	RcHm3g0472961	probable purple acid phosphatase 20
530	RcHm3g0472971	LITTLE ZIPPER 4
531	RcHm3g0472981	CAAX prenyl protease 2
532	RcHm3g0472991	YIPF5 homolog
533	RcHm3g0473001	PREDICTED: uncharacterized protein LOC105352157
534	RcHm3g0473011	TFL1
535	RcHm3g0473021	TERMINAL FLOWER 1
536	RcHm3g0473031	Alpha beta-Hydrolases superfamily
537	RcHm3g0473041	germin subfamily 3 member 2
538	RcHm3g0473061	Unknown function
539	RcHm3g0473071	ABSCISIC ACID-INSENSITIVE 5
540	RcHm3g0473081	YLS9-like
541	RcHm3g0473091	YLS9-like
542	RcHm3g0473101	YLS9-like
543	RcHm3g0473111	YLS9-like
544	RcHm3g0473121	YLS9-like
545	RcHm3g0473131	F-box LRR-repeat At1g67190-like
546	RcHm3g0473141	Unknown function
547	RcHm3g0473151	Unknown function
548	RcHm3g0473161	POLYCHOME-like
549	RcHm3g0473171	iron-sulfur assembly -like mitochondrial
550	RcHm3g0473181	POLYCHOME-like
551	RcHm3g0473191	cell division homolog 2- chloroplastic-like
552	RcHm3g0473201	pentatricopeptide repeat-containing At4g21065-like
553	RcHm3g0473211	probable disease resistance At4g27220
554	RcHm3g0473221	peptidyl-prolyl cis-trans isomerase PASTICCINO1-like
555	RcHm3g0473231	cell division homolog 2- chloroplastic-like
556	RcHm3g0473241	Unknown function
557	RcHm3g0473251	peroxidase 64
558	RcHm3g0473261	pentatricopeptide repeat-containing At4g21065-like
559	RcHm3g0473271	pentatricopeptide repeat-containing At4g21065-like
560	RcHm3g0473281	Unknown function
561	RcHm3g0473291	probable disease resistance At4g27220
562	RcHm3g0473301	peptidyl-prolyl cis-trans isomerase PASTICCINO1-like
563	RcHm3g0473311	cell division homolog 2- chloroplastic-like
564	RcHm3g0473321	pentatricopeptide repeat-containing At4g21065-like
565	RcHm3g0473331	pentatricopeptide repeat-containing At4g21065-like
566	RcHm3g0473341	probable disease resistance At4g27220
567	RcHm3g0473351	cell division homolog 2- chloroplastic-like
568	RcHm3g0473361	pentatricopeptide repeat-containing At4g21065-like
569	RcHm3g0473371	pentatricopeptide repeat-containing At4g21065-like
570	RcHm3g0473381	probable disease resistance At4g27220
571	RcHm3g0473391	probable disease resistance At4g27220
572	RcHm3g0473401	cell division homolog 2- chloroplastic
573	RcHm3g0473411	nuclease HARBI1
574	RcHm3g0473421	Unknown function
575	RcHm3g0473431	pentatricopeptide repeat-containing At4g21065-like
576	RcHm3g0473441	probable disease resistance At4g27220
577	RcHm3g0473471	pentatricopeptide repeat-containing At4g21065-like
578	RcHm3g0473481	probable disease resistance At4g27220
579	RcHm3g0473491	cell division homolog 2- chloroplastic-like
580	RcHm3g0473501	pentatricopeptide repeat-containing At4g21065-like
581	RcHm3g0473511	pentatricopeptide repeat-containing At4g21065-like
582	RcHm3g0473521	Unknown function
583	RcHm3g0473531	probable disease resistance At4g27220
584	RcHm3g0473541	Unknown function
585	RcHm3g0473551	pentatricopeptide repeat-containing At4g21065-like
586	RcHm3g0473561	probable disease resistance At4g27220
587	RcHm3g0473571	probable disease resistance At4g27220
588	RcHm3g0473581	PREDICTED: uncharacterized protein LOC105352154
589	RcHm3g0473591	transcription factor RAX2-like
590	RcHm3g0473601	thylakoid lumenal 19 kDa chloroplastic
591	RcHm3g0473611	probable cytosolic iron-sulfur assembly CIAO1 homolog
592	RcHm3g0473621	cytochrome b-c1 complex subunit 9-like
593	RcHm3g0473631	YLS9-like
594	RcHm3g0473641	YLS9-like
595	RcHm3g0473651	60S ribosomal L10a-1-like
596	RcHm3g0473661	iron-sulfur assembly -like mitochondrial
597	RcHm3g0473671	glucosamine 6-phosphate N-acetyltransferase-like
598	RcHm3g0473681	Syntaxin t-SNARE family
599	RcHm3g0473691	Iron-sulfur cluster biosynthesis family isoform 1
600	RcHm3g0473701	F-box FBD LRR-repeat At1g13570-like
601	RcHm3g0473711	iron-sulfur cluster biosynthesis family partial
602	RcHm3g0473721	iron-sulfur cluster biosynthesis family partial
603	RcHm3g0473731	Iron-sulfur cluster biosynthesis family isoform 1
604	RcHm3g0473741	Unknown function
605	RcHm3g0473751	tropinone reductase homolog At1g07440-like
606	RcHm3g0473761	Iron-sulfur cluster biosynthesis family isoform 1
607	RcHm3g0473771	aspartic ase CDR1-like
608	RcHm3g0473781	aspartic ase CDR1-like
609	RcHm3g0473791	pentatricopeptide repeat-containing At2g36240
610	RcHm3g0473801	1-(5-phosphoribosyl)-5-[(5-phosphoribosylamino)methylideneamino] imidazole-4-carboxamide chloroplastic
611	RcHm3g0473811	heme-binding chloroplastic
612	RcHm3g0473821	PREDICTED: uncharacterized protein LOC105352151
613	RcHm3g0473831	PREDICTED: uncharacterized protein LOC101292431
614	RcHm3g0473841	alpha carbonic anhydrase chloroplastic-like
615	RcHm3g0473861	PREDICTED: uncharacterized protein At3g28850
616	RcHm3g0473871	Unknown function
617	RcHm3g0473881	F-box kelch-repeat At1g57790-like
618	RcHm3g0473891	heterogeneous nuclear ribonucleo R
619	RcHm3g0473901	replication A 14 kDa subunit B
620	RcHm3g0473911	F-box LRR-repeat At4g14103-like
621	RcHm3g0473921	Unknown function
622	RcHm3g0473931	transparent testa
623	RcHm3g0473941	laccase-15-like
624	RcHm3g0473951	F-box LRR-repeat At4g14103-like
625	RcHm3g0473961	F-box LRR-repeat At4g14103-like
626	RcHm3g0473971	probable disease resistance At5g66900
627	RcHm3g0473981	Unknown function
628	RcHm3g0473991	F-box kelch-repeat At1g57790-like
629	RcHm3g0474001	F-box LRR-repeat At4g14103-like isoform X1
630	RcHm3g0474011	F-box At5g03100-like
631	RcHm3g0474021	F-box FBD LRR-repeat At4g03220

Supplementary Table 2. Rose cultivars used in this study.

Phenotype	Rose species/variety	Code
Simple flower roses	*R. wichurana*	WIC
	R. gigantea	GIG
	R. chinensis 'Spontanea'	SPO
	R. chinensis 'Sanguinea'	SAN
	R. chinensis 'Mutabilis'	MUT
	R. moschata	MOS
Double Flower roses	*R. chinensis* 'Old Blush'	OB
	R. x hybrida 'Rouge Meilland'	RME
	R. x hybrida 'La France'	FRA
	R. odorata 'Hume's Blush'	HUM
	R. x hybrida 'Bébé Fleuri'	BFL
	R. x hybrida 'Bengale d'Automne'	BENG
	R. x hybrida 'Cramoisi Supérieur'	CRAM
	R. x hybrida 'Comtesse de Cayla'	COMT
	R. x hybrida 'Ducher'	DUS
	R. x hybrida 'General Shablikine'	GEN
	R. x hybrida 'Blush Noisette'	NOIS
	R. x hybrida 'Hérodiade'	HERO
	R. x hybrida 'Louise d'Arzens'	LOUI

Supplementary Table 3. Primers used in this study.

Genotyping primers	RcAP2L_TE_left_border_F	GTGGTGAGAAGTAGTTCCTAGCTAG
	RcAP2L_TE_left_border_R	AGACCAAATAGTAGATCCTCACACG
	RcAP2L_TE_right_border_F	GAAGCTGCAAGGTCTTTACGTTAAT
	RcAP2L_TE_right_border _R	GAGACTCTTCCACTTTGTTTCCTTG
	RcAP2Lwt_F	ATTGCTATGTCATGTCTTCACAACC
	RcAP2Lwt_R	TATGGATGGGAAAGAAGTTGGAGTT
cDNA sequencing primers	RcAP2L_F	CACCATGCTAGATCTCAACCTCAA
	RcAP2LΔ172_R	TTATTTGCTTCGGAAAACCC
	RcAP2Lwt_R	TCAGCTCCTGCAATAGAAAT

Supplementary Data File 1

>At-SMZ
VKKSRRGPRSRSSQYRGVTFYRRTGRWESHIWDCGKQVYLGGFDTAYAAARAYDRAAIKF
RGLDADINFVVDDYRHDIDKMKNLNKVEFVQTLRRESASFGRGSSKYKGLALQKCTQFKT
HDQIHLFQNRGWDAAAIKYNELGKGEGAMKFGAHIKGNGHNDLELSLGI
>Carubv10018526m
VKKSRRGPRSRSSQYRGVTFYRRTGRWESHIWDCGKQVYLGGFDTAYAAARAYDRAAIKF
RGLDADINFVVDDYRHDIDKMKNLNKVEFVQTLRRESASFGRGSSKYKGLALQKCTQFKT
HHDQIHLFQNRGWDAAALKYNELGKGEGGMKFGAHIK
>At-SNZ
KKKSRRGPRSRSSHYRGVTFYRRTGRWESHIWDCGKQVYLGGFDTAYTAARAYDRAAIRF
RGLQADINFIVDDYKQDIEKMKNLSKEEFVQSLRRASASLARGGSKYKNTHMRNDHIHLF
QNRGLNAAAAKCNEIRKMEGDIKLGAHSKGNEHNDLELSLGI
>Carubv10023614m
KKKSRRGPRSRSSNYRGVTFYRRTGRWESHIWDCGKQVYLGGFDTAYTAARAYDRAAIKF
RGLQADINFIVDDYKQDFEKMKNLSKEEFVQTLRRASASLARGGSKYKNNYMKNDYIHLF
QNRQWNASAAKCNEIRKIEAGEIKLGAHHNKGNEHKDLDLSLGI
>At-TOE2
VKKSRRGPRSKSSQYRGVTFYRRTGRWESHIWDCGKQVYLGGFDTAHAAARAYDRAAVKF
RGLEADINFVIGDYEEDLKQMANLSKEEVVQVLRRQSSGFSRNNSRYQGVALQKIGGWGA
QMEQLHGNMGCDKAAVQWKGREAASLIEPHASRMIPEAANVKLDLNLGI
>Carubv10026292m
VKKSRRGPRSKSSQYRGVTFYRRTGRWESHIWDCGKQVYLGGFDTAHAAARAYDRAAVKF
RGLEADINFIISDYEEDLKQMANLSKEEVVQVLRRQSSGFSRNNSRYQGVALQKIGGWGA
QMEQFHGNMACDKAAIQWNGRQAASFIEPHGSRMIPEAANVKLDLNLGI
>Medtr1g049140.1
VRKNRRGPRSRSSQYRGVTFYRRTGRWESHIWDCGKQVYLGGFDTAHAAARAYDRAAVMF
RGVDADINFNLSDYEDLKQMRGLSKEEFVLRLRRKTNENSKRSSTYRRTLSLNKYGQGDP
QIITPFVAKEFCHKSPIKRDHEVETSCKPSSIYKGEIVANSNKTGTLHNLDLSLGI
>Prupe.2G220100.1.p
PRKSRRGPRSRSSQYRGVTFYRRTGRWESHIWDCGKQVYLGGFDTAHSAARAYDRAAIKF
RGVDADINFTLGDYEEDMKQLGHLNKEEFVHVLRRQSTGASRGNSKYRGVALPKCGGAGG
RWEARMAQFPEKKVFEKEGIKYNTGREAAAVTNFVDPSIYEGEVVLDASIEGSGHNLDLS
LGI
>RcHm1g0364341
ARKSRRGPRSRSSQYRGVTFYRRTGRWESHIWDCGKQVYLGGFDTAHSAAKAYDRAAIKF
RGVDADINFVLSDYEEDLKQMRHLNKEEFVHVLRRQSNGASRGNSKLRGVAAAAAAAAAV
PKIGPFEVRMGQLIPGNKLIKCNGREGVANFDPSIYEGAVVVDASTEGSGHNLDLSLGI
>Fv-gene23252-v1.0-ab_initio
ARKSRRGPRSRSSQYRGVTFYRRTGRWESHIWDCGKQVYLGGFDTAHSAAKAYDRAAIKF
RGVDADINFVLSDYEEDLKQMRHLNKEEFVHVLRRQSNGASRGNSKFRGVAAAAATAAAM
PKIGPFEVHMGQLIPGNKLIKGNGREGLSNFDPSIYEGEVVVGASTEGSGHNLDLSLGI
>Peaxi162Scf01024g00326.1
AKKSRRGPRSRSSQYRGVTFYRRTGRWESHIWDCGKQVYLGGFDTAHAAARAYDRAAIKF
RGVDADINFSITDYEEDMKQMKNLSKEEFVQILRRQSTGFSRGNSKFRGVTLQKCGRWEG
RMGQFLGKKDYDKAAITCNGREAISNFQPNTYGREIKEDNKDGGSGENLDLNLWI
>Solyc04g049800.2.1
HKKSRRGPRSRSSQYRGVTFYRRTGRWESHIWDCGKQVYLGGFDTAHAAARAYDRAAIKF
RGVDADINFNITDYNEDMKQMKNLSKEEFVQVLRRHSNGFSRGNSKYRGVTLHKCGRWEG
QFIGKKAYDKCDGREAVTNFVASTYEREKKEDKRDGGSGENLDLNLWI
>GSVIVT01016352001
RRGPRSRSSQYRGVTFYRRTGRWESHIWDCGKQVYLGGFDTAHAAARAYDRAAIKFRGVD
ADINFSISDYEEDMKQMKNLNKEEFVHILRRQSNGFSRGSSKYRGVTLHKCGRWEARMGQ
FLGKKYIYLGLFDSEIEAARAYDKAAIRYNGREAVTNFVPSTYGEGAILEANDRSSGHNL
DLNLGL
>Peaxi162Scf00072g00229.1*
VKKKSRRGPRSRSSQYRGVTFYRRTGRWESHIWDCGKQVYLGGFDTADVAARAYDRAAIK

FRGVDADINFSITDYEEDMKQLKHLGKEEFVHVLRRQSNGFSRGSSKFRGVTLHKCGRWE
ARMGQFLGKKYIYLGLFDSEVEAARAYDKAAIKTNGREAVTNFEPSAYEGETISEPQSEG
SHHNLDLNLGI
>Sl-AP2e
VKKSRRGPRSRSSQYRGVTFYRRTGRWESHIWDCGKQVYLGGFDTAHTAARAYDRAAIKF
RGVDADINFSLSDYEEDMQQMKNLGKEEFVHLLRRHSTGFSRGSSKFRGVTLHKCGRWEA
RMGQFLGKKYIYLGLFDSEVEAARAYDKAAIKTSGREAVTNFEPSSYEGETMSLPQSEGS
QHDLDLNLGI
>Peaxi162Scf00389g00028.1*
VKKSRRGPRSRSSQYRGVTFYRRTGRWESHIWDCGKQVYLGGFDTAHAAARAYDRAAIKF
RGVDADINFNLSDYDEDMKQMKNLSKEEFVHMLRRQSTGFSRGSSKYRGVTLHKCGRWEA
RMGQFLGKKYIYLGLFDSEVEAARAYDKAAIKCNGREAVTNFEPSTYEGETISDPRSEGS
QQNLDLNLGI
>Sl-AP2d
VKKSRRGPRSRSSQYRGVTFYRRTGRWESHIWDCGKQVYLGGFDTAHAAARAYDRAAIKF
RGVDADINFNLSDYEEDMKQMKNLSKEEFVHVLRRQSTGFSRGSSKYRGVTLHKCGRWEA
RMGQFLGKKYIYLGLFDSEVEAARAYDKAAIKCNGREAVTNFEPSAYEGEINSNPQSEGS
QQDLDLNLGI
>Prupe.6G091100.1.p
VKKSRRGPRSRSSQYRGVTFYRRTGRWESHIWDCGKQVYLGGFDTAHAAARAYDRAAIKF
RGVDADINYNLSDYEEDLKQMKNLTKEEFVHILRRQSTGFSRGSSRYRGVTLHKCGRWEA
RMGQFLGKKYIYLGLFDSEVEAARAYDKAAIKCNGREAVTNFEPSTYEGEMISEAGNEDG
DHNLDLNLGI
>GSVIVT01025100001
VKKSRRGPRSRSSQYRGVTFYRRTGRWESHIWDCGKQVYLGGFDTAHAAARAYDRAAIKF
RGVDADINFNLSDYDEDLKQMKNLTKEEFVHILRRQSTGFSRGSSKYRGVTLHKCGRWEA
RMGQFLGKKYIYLGLFDSEVEAARAYDKAAIKCNGREAVTNFEPSTYEGEMIPEASNEGS
DHNLDLNLGI
>Medtr2g093060.1
AKKSRRGPRSRSSQYRGVTFYRRTGRWESHIWDCGKQVYLGGFDTAHAAARAYDRAAIKF
RGLDADINFNLVEYEEDMNQMKNLSKEEFVHILRRHSNGFSRGSSKYRGVTLHKCGRWEA
RMGQLLGKKYIYLGLFDSEVEAARAYDKAALKCNGREAVTNFEPSTYENEMKPGAINEGG
SHNLDLNLGM
>Medtr4g061200.2
VKKSRRGPRSRSSQYRGVTFYRRTGRWESHIWDCGKQVYLGGFDTAHAAARAYDRAAIKF
RGVGADINFNLNDYDDDLKQTKNLSKEEFVQILRRQSNGFSRGSSKYRGVTLHKCGRWEA
RMGQFLGKKYIYLGLFDSEVEAARAYDKAAIQNNGREAMTNFEASTYEGEMKSAAINEGG
SQNLDLNLGI
>At-TOE1
VKKSRRGPRSRSSQYRGVTFYRRTGRWESHIWDCGKQVYLGGFDTAHAAARAYDRAAIKF
RGVDADINFTLGDYEEDMKQVQNLSKEEFVHILRRQSTGFSRGSSKYRGVTLHKCGRWEA
RMGQFLGKKYIYLGLFDSEVEAARAYDKAAINTNGREAVTNFEMSSYQNEINSESNNSEI
DLNLGI
>Carubv10025387m
VKKSRRGPRSRSSQYRGVTFYRRTGRWESHIWDCGKQVYLGGFDTAHAAARAYDRAAIKF
RGVDADINFTLGDYEEDMKQVQNLSKEEFVHILRRQSTGFSRGSSKYRGVTLHKCGRWEA
RMGQFLGKKYIYLGLFDSEVEAARAYDKAAINTNGREAVTNFEMSSYQNEINSETNNEGG
HDKLDLNLGM
>Ph-BEN
VKKSRRGPRSKSSQYRGVTFYRRTGRWESHIWDCGKQVYLGGFDTAHAAARAYDRAAIKF
RGLDADINFNVSDYQDDLKQMTNFTKEEFVHILRRQSTGFSRGSSQYRGVTLHKCGRWES
RMGQFLGKKYIYLGLFDSEIEAARAYYKAAIKCNGREAVTNFELSTYEGELSTEADNGGA
SHNLDLNLGI
>Solyc09g007260.2.1
VKKSRRGPRSRSSQYRGVTFYRRTGRWESHIWDCGKQVYLGGFDTAHAAARAYDRAAIKF
RGLDADINFNVSDYHDDLKQMGNFSKEEFVHILRRQSTGFSRGSSKYRGVTLHKCGRWEA
RMGQFLGKKAYDKAAIKCNGKEAVTNFELSTYEGELSTEAENGGADHNLDLNLGI
>Ph-BOB

IKKSRRGPRSRSSQYRGVTFYRRTGRWESHIWDGGKQVYLGGFDTAHAAARAYDRAAIKF
RGLDADINFNVSDYEEDLKQMKNFSKEEFLHILRRQSTGFSRGSSKFRGVTLHKCGRWEA
RMGQLLGKKYIYLGLFDSEIEAARAYDKAAIKSNGREAVTNFELSTYEGVLSSETADIGG
TSHNLDLRLGI
>Solyc10g084340.1.1
KKSRRGPRSRSSQYRGVTFYRRTGRWESHIWDCGKQVYLGGFDTAHAAARAYDRAAIKFR
GLDADINFNISDYEEDLKQMKNFSKEEFVHILRRQSNGFSRGSSKYRGVTLHKCGRWEAR
MGQLLGKKYIYLGLFDNEIEAARAYDKAAIKCNGREAVTNFEPNTYEEALSSEADTGGTD
HNIDLNLGI
>Medtr7g100590.1
VKKSRRGPRSRSSQYRGVTFYRRTGRWESHIWDCGKQVYLGGFDTAHAAARAYDRAAIKF
RGVDADINFNVSDYDEDIKQMNNFTKEEFVHILRRQSTGFSRGSSKYRGVTLHKCGRWEA
RMGQFLGKKYIYLGLFDSELDAARAYDKAAIKCNGREAVTNFEASSYEGELTSQADNDDI
KQNLDLNLGI
>Prupe.6G242400.1.p
VKKSRRGPRSRSSQYRGVTFYRRTGRWESHIWDCGKQVYLGGFDTAHAAARAYDRAAIKF
RGTEADINFNVSDYEDDIKQMSNFTKEEFVHILRRQSTGFSRGSSKYRGVTLHKCGRWEA
RMGQFLGKKYIYLGLFDSEIEAARAYDKAAIKCNGREAVTNFEPNSYDGQIMSEAHNGGS
DKSLDLNLGI
>GSVIVT01025548001
VKKSRRGPRSRSSQYRGVTFYRRTGRWESHIWDCGKQVYLGGFDTAHAAARAYDRAAIKF
RGADADINFAVSDYDEDIKQMSNFTKEEFVHVLRRGSTGFSRGSSKYRGVTLHKCGRWEA
RMGQFLGKKYIYLGLFDSEIEAARAYDKAAIKCNGREAVTNFEPSTYEGEIIFPADNGGS
SQNLNLLPNLDLHLGV
>Ph-ROB1
MKKSRRGPRSRSSQYRGVTFYRRTGRWESHIWDCGKQVYLGGFDTAHAAARAYDRAAIKF
RGVEADINFNLEDYEGDLKQMTNLTKEEFVHVLRRQSTGFPRGSSKYRGVTLHKCGRWEA
RMGQFLGKKYVYLGLFDTEVEAARAYDKAAIKCNGKDAVTNFDPSIYENELNSTESTDSG
ADHNLDLSLGG
>Sl-AP2c
IKKSRRGPRSRSSQYRGVTFYRRTGRWESHIWDCGKQVYLGGFDTAHAAARAYDRAAIKF
RGVEADINFSLEDYESDLKQMTSLTKEEFVHVLRRQSTGFPRGSSKYRGVTLHKCGRWEA
RMGQFLGKKYVYLGLFDTEIEAARAYDKAAIKCNGKDAVTNFDPSIYENELNSIESTDNV
ADHNLDLSLGG
>Ph-ROB2
VKKSRRGPRSRSSQYRGVTFYRRTGRWESHIWDCGKQVYLGGFDTAHAAARAYDRAAIKF
RGVEADINFTLEDYEEDLKQMTNLTKEEFVHVLRRQSTGFPRGSSKYRGVTLHKCGRWEA
RMGQFLGKKYVYLGLFDTEVEAARAYDKAAIKCNGKDAVTNFDRSIYENELNTTEFTDNA
ADHNLDLSLGS
>Sl-AP2a
IKKSRRGPRSRSSQYRGVTFYRRTGRWESHIWDCGKQVYLGGFDTAHAAARAYDRAAIKF
RGAEADINFTSKDYEDDLKQMSNLTKEEFVHVLRRQSTGFPRGSSKYRGVTLHKCGRWEA
RMGQFLGKKYVYLGLFDTEVEAARAYDKAAIKCNGKDAVTNFDRSIYENELNSTECTDNA
TDHNLDLSLGG
>Ph-ROB3
MKKSRRGPRSRSSQYRGVTFYRRTGRWESHIWDCGKQVYLGGFDTAHAAARAYDRAAIKF
RGVEADINFNLEDYEEDLKQMRNLTKEEFVHVLRRQSTGFPRGSSKYRGVTLHKCGRWEA
RMGQLLGKKYVYLGLFDTEVEAARAYDKAAIKCNGKDAVTNFDSSIYGNELNLTESTHKA
LDHSLDLSLGG
>Sl-AP2b
MKKSRRGPRSRSSQYRGVTFYRRTGRWESHIWDCGKQVYLGGFDTAHAAARAYDRAAIKF
RGMEADINFNLEDYEEDLKQMKNLTKEEFVHVLRRQSTGFPRGSSKYRGVTLHKCGRWEA
RMGQLLGKKYVYLGLFDTENEAARAYDKAAIKCNGKDAVTNFDPCIYENELNSSECSNKA
ADHSLDLSLGS
>GSVIVT01022081001
LKKSRRGPRSRSSQYRGVTFYRRTGRWESHIWDCGKQVYLGGFDTAHAAARAYDRAAIKF
RGVEADINFSLEDYEEDLKQMGNLTKEEFVHVLRRQSTGFPRGSSKYRGVTLHKCGRWEA
RMGQFLGKKYVYLGLFDTEIEAARAYDKAAIKCNGKEAVTNFDPSIYENELNSSGNAADH

NLDLSLGG
>Medtr5g016810.1
MKKSRRGPRSRSSQYRGVTFYRRTGRWESHIWDCGKQVYLGGFDTAHAAARAYDRAAIKF
RGVEADINFNIEDYEEDLKQMSNLTKEEFVHVLRRQSTGFPRGSSKYRGVTLHKCGRWEA
RMGQFLGKKYVYLGLFDTEIEAARAYDKAAIKCNGKEAVTNFDPSIYDNELNSESSGNAA
DHNLDLSLGN
>Prupe.6G231700.1.p
MKKSRRGPRSRSSQYRGVTFYRRTGRWESHIWDCGKQVYLGGFDTAHAAARAYDRAAIKF
RGVEADINFSIEDYEEDLKQMTNLTKEEFVHVLRRQSTGFPRGSSKYRGVTLHKCGRWEA
RMGQFLGKKYVYLGLFDTEIDAARAYDKAAIKCNGKEAVTNFDPSIYENELNPSSESSGV
NAAEHNLDLSLGS
>RcHm2g0106221
MKKSRRGPRSRSSQYRGVTFYRRTGRWESHIWDCGKQVYLGGFDTAHAAARAYDRAAIKF
RGVEADINFNIDDYEEDLKQMSNLTKEEFVHVLRRQSTGFPRGSSKYRGVTLHKCGRWEA
RMGQFLGKKYVYLGLFDTEIDAARAYDKAAIKCNGKEAVTNFDPSIYESELNSTEPSSIN
AGDHNLDLSLGN
>Fv-gene23806-v1.0-ab_initio
MKKSRRGPRSRSSQYRGVTFYRRTGRWESHIWDCGKQVYLGGFDTAHAAARAYDRAAIKF
RGVEADINFNIEDYEEDLKQMSNLTKEEFVHVLRRQSTGFPRGSSKYRGVTLHKCGRWEA
RMGQFLGKKYVYLGLFDTEIDAARAYDKAAIKCNGKEAVTNFDPSIYESELNTTEPASGN
NNAADHNLDLSLGN
>Medtr4g094868.1
MKKSRRGPRSRSSQYRGVTFYRRTGRWESHIWDCGKQVYLGGFDTAHAAARAYDRAAIKF
RGVEADINFNIEDYEEDLKQMTNLTKEEFVHVLRRQSTGFPRGSSKYRGVTLHKCGRWEA
RMGQFLGKKYVYLGLFDTEVEAARAYDKAAIKCNGKEAVTNFDPSIYDSELNNSDPASNA
GDHNLDLSLGN
>At-AP2
LKKSRRGPRSRSSQYRGVTFYRRTGRWESHIWDCGKQVYLGGFDTAHAAARAYDRAAIKF
RGVEADINFNIDDYDDDLKQMTNLTKEEFVHVLRRQSTGFPRGSSKYRGVTLHKCGRWEA
RMGQFLGKKYVYLGLFDTEVEAARAYDKAAIKCNGKDAVTNFDPSIYDEELNAESSGNPT
TPQDHNLDLSLGN
>Carubv10004818m
LKKSRRGPRSRSSQYRGVTFYRRTGRWESHIWDCGKQVYLGGFDTAHAAARAYDRAAIKF
RGVEADINFNIEDYDDDLKQMTNLTKEEFVHVLRRQSTGFPRGSSKYRGVTLHKCGRWEA
RMGQFLGKKYVYLGLFDTEVEAARAYDKAAIKCNGKDAVTNFDPSIYDEELNAESSGNHP
TPQDHNLDLSLGN
>At-TOE3
IKKSRRGPRSRSSQYRGVTFYRRTGRWESHIWDCGKQVYLGGFDTAHAAARAYDRAAIKF
RGVDADINFDIEDYLDDLKQMGNLTKEEFMHVLRRQSTGFPRGSSKYRGVTLHKCGRWES
RLGQFLNKKYVYLGLFDTEIEAARAYDKAAIKCNGKDAVTNFDPKVYEEEEDLSSETTRN
GHNLGLSLGE
>Carubv10026667m
LKKSRRGPRSRSSQYRGVTFYRRTGRWESHIWDCGKQVYLGGFDTAHAAARAYDRAAIKF
RGADADINFDIEDYMDDLKQMGNLTKEEFMHVLRRQSTGFPRGSSKYRGVTLHKCGRWES
RLGQFLNKKYVYLGLFDTEIEAARAYDKAAIKCNGKDAVTNFDPKVYEEEDLIPEMKWNG
HNLDLSLGE
>RcHm5g0061501
VKKSRRGPRSRSSQYRGVTFYRRTGRWESHIWDCGKQVYVGGFDTAHAAARAYDRAAIKF
RGVDADINYNLSDYEEDMKQMKNLTKEEFVHILRRQSTGFSRGSSRYRGVTLHKCGRWEA
RMGQFLGKKYIYLGLFDSEVEAARAYDKAAIKCNGREAVTNFEASTYEGEMISDAVNEDG
NHNLDLNLGI
>Fv-gene25362-v1.0-ab_initio
VKKSRRGPRSRSSQYRGVTFYRRTGRWESHIWDCGKQVYLGGFDTAHAAARAYDRAAIKF
RGVDADINYNLSDYEEDMKQMKNLTKEEFVHILRRQSTGFSRGSSKYRGVTLHKCGRWEA
RMGQFLGKKYIYLGLFDSEVEAARAYDKAAIKCNGREAVTNFEASTYEGEMISDAGNEDG
NHNLDLNLGI
>RcHm3g0468481
VKKSRRGPRSRSSQYRGVTFYRRTGRWESHIWDNGKQVYLGGFDTAHSAARAYDRAAIKF

RGTEADINFNVSDYDEDIKQMSKFTKEEFVHILRRQSTGFARGSSKYRGVTLHKCGRWEA
RMGQLLGKKYIYLGLFDSEIEAARAYDQAAIKCHGREAVTNFNPSLYDGEIMAEANNAGI
DDSLDLNLGI
>RcHt_S350.36
VKKSRRGPRSRSSQYRGVTFYRRTGRWESHIWDNGKQVYLGGFDTAHSAARAYDRAAIKF
RGTEADINFNVSDYDEDIKQMSKFTKEEFVHILRRQSTGFARGSSKYRGVTLHKCGRWEA
RMGQLLGKKYIYLGLFDSEIEAARAYDQAAIKCHGREAVTNFNPSLYDGEIMAEANNAGI
DDSLDLNLGI
>Fv-gene18070-v1.0-ab_initio
VKKSRRGPRSRSSQYRGVTFYRRTGRWESHIWDNGKQVYLGGFDTAHSAARAYDRAAIKF
RGTEADINFSVGDYDEDIKQMSNYTKEEFVQILRRQSTGFARGSSKYRGVTLHKCGRWEA
RMGQLLGKKYIYLGLFDTEIEAARAYDQAAIKCHGSEAVTNFNPILYDGEIEAEPNNAGM
DGSLDLNLGI
>Ph-ANT1
HRKSIDTFGQRTSQYRGVTRHRWTGRYEAHLWDNSCKKEGQSRKGRQVYLGGYDMEEKAA
RAYDLAALKYWGPSTHINFPLENYQKELEDMNNMTRQEYVAHLRRKSSGFSRGASMYRGV
TRHHQHGRWQARIGRVAGNKDLYLGTFSTQEEAAEAYDVAAIKFRGANAVTNFDMSRYEV
ERIMASNTLLSGELARRKKDT
>Ph-ANT2
HRKSIDTFGQRTSQYRGVTRHRWTGRYEAHLWDNSCKKEGQTRKGRQVYLGGYDMEEKAA
RAYDLAALKYWGPSTHINFPLENYQKELEEMKNMTRQEYVAHLRRKSSGFSRGASMYRGV
TRHHQHGRWQARIGRVAGNKDLYLGTFSTQEEAAEAYDVAAIKFRGVNAVTNFDISRYDV
EKIMASNTLPAGEFARRTKER
>At-ANT
HRKSIDTFGQRTSQYRGVTRHRWTGRYEAHLWDNSFKKEGHSRKGRQVYLGGYDMEEKAA
RAYDLAALKYWGPSTHTNFSAENYQKEIEDMKNMTRQEYVAHLRRKSSGFSRGASIYRGV
TRHHQHGRWQARIGRVAGNKDLYLGTFGTQEEAAEAYDVAAIKFRGTNAVTNFDITRYDV
DRIMSSNTLLSGELARRNNNS
